# The Hippo pathway in cancer: YAP/TAZ and TEAD as therapeutic targets in cancer

**DOI:** 10.1042/CS20201474

**Published:** 2022-02-04

**Authors:** Richard Cunningham, Carsten Gram Hansen

**Affiliations:** University of Edinburgh Centre for Inflammation Research, Institute for Regeneration and Repair, Queen’s Medical Research Institute, Edinburgh bioQuarter, 47 Little France Crescent, Edinburgh EH16 4TJ, U.K.

**Keywords:** AlphaFold, Cancer, Hippo pathway, Immuno-oncology, Mesothelioma, Sarcoma

## Abstract

Tumorigenesis is a highly complex process, involving many interrelated and cross-acting signalling pathways. One such pathway that has garnered much attention in the field of cancer research over the last decade is the Hippo signalling pathway. Consisting of two antagonistic modules, the pathway plays an integral role in both tumour suppressive and oncogenic processes, generally via regulation of a diverse set of genes involved in a range of biological functions. This review discusses the history of the pathway within the context of cancer and explores some of the most recent discoveries as to how this critical transducer of cellular signalling can influence cancer progression. A special focus is on the various recent efforts to therapeutically target the key effectors of the pathway in both preclinical and clinical settings.

## Introduction

The Hippo pathway is an evolutionarily conserved signalling cascade regulated by a broad spectrum of upstream effectors and acts as an integral mechanosensory component of cells, transducing physical signals at the plasma membrane and regulating response via control of differentiation and proliferation in cells [1–3]. The pathway was initially discovered in *Drosophila melanogaster* following identification of core genes regulating organ size control and cellular overgrowth via mosaic screening. Genes identified include Warts (*Wts*, *LATS1/2* in human) [4,5] and Hippo (*Hpo*, or *STK4/3* encoding MST1/2 in human) [6,7], which encode the kinases that constitute the phosphorylation cascade central to the Hippo pathway, which is a central regulator of early stage development in embryogenesis. This role is evidenced by the early embryonic lethality observed when the primary, downstream transcriptional activators of the pathway, Yap (encoded by *Yap1*) and Taz (encoded by* Wwtr1)*, are both lost in mice [8]. In such cases, embryos fail to progress past morula stage, likely due to the role both gene products play in lineage specification during embryogenesis. Moreover, just homozygous loss of *Yap* results in extreme morphological disruption in mouse embryos and an inability to survive beyond embryonic day 10.5 [9]. The Hippo gene (*Hpo*) was named due to the highly proliferative overgrowth phenotype upon loss-of-function mutation of the *Hpo* gene and consequently observed increased head size in the *Drosophila*; this phenotype somewhat resembled a Hippo head under the microscope [6,7,10]. A similar, general effect is observed on loss-of-function mutations of the other core kinase cascade members. These kinases and scaffolding components act as the primary regulators of the Hippo signalling pathway, whose activities are involved in a variety of oncogenic processes and pathways [11,12]. Its close involvement with regenerative and pro-cancerous functions have made the Hippo signalling pathway an attractive, although challenging, target for drug discovery efforts of late [13].

**Figure 1:**
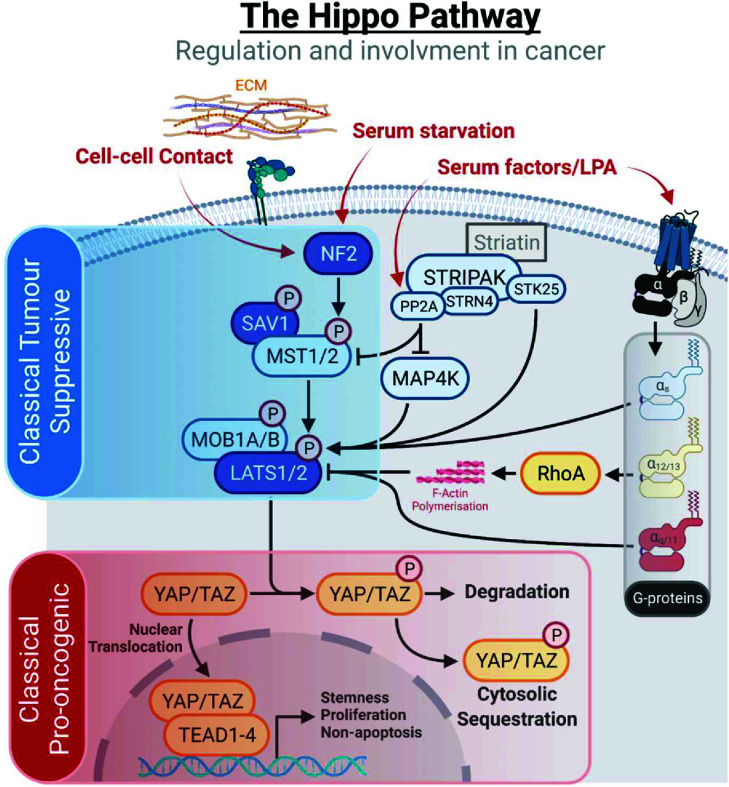
The Hippo pathway consists of distinct oncogenic and tumour suppressive modules Schematic of the core Hippo pathway, including the generally tumour suppressive core kinase module (highlighted in blue) and tumorigenic transcriptional module (highlighted in red). A selection of upstream, regulatory components are additionally included. Protein products of genes frequently mutated in various specific tumour types are shown in darker colours. Note that MST1/2 are encoded by *STK4/3* respectively and TAZ by *WWTR1*.

The Hippo pathway itself consists of two primary modules: the core serine/threonine kinase cascade, which is modulated by a variety of upstream signals, and the transcriptional module that drives the expression of downstream target genes (Figure 1). Throughout the course of vertebrate evolution, duplication events have resulted in multiple paralogues of various Hippo family components [14,15]. The classical core kinase cascade consists of MST1/2 (Hippo or Hpo) and its binding partner SAV1 (Salvador), which act to phosphorylate and activate LATS1/2 (Warts or Wts) and its binding partners MOB1A/B (Mob) [16]. Multiple additional upstream components of the kinase cascade have been described, with examples including KIBRA and NF2 (Merlin) that form spatially regulatory and kinase activating scaffolds that consequently increase LATS/MST activity [17–19]. More recently, the mitogen-activated kinase kinase kinase kinase (MAP4K) family of kinases, which act as upstream activators of the generally tumorigenic MAPK pathway [20], have also been shown to play an integral role in activating the Hippo kinase cascade, directly phosphorylating and activating LATS1/2, in parallel with the Hippo kinases MST1/2 [21]. This interaction is regulated by STRN4 (or zinedin) [22,23], a member of the striatin-interacting phosphatase and kinase (STRIPAK) complexes, which act as general regulators of a range of pathways active in cancer development [24]. The STRIPAK complex is a heterogeneous and adaptable core complex with a central regulatory role within the Hippo pathway [3], acting to modulate the activity of Hippo pathway kinases at multiple levels [25] beyond just through MAP4K-mediated cascade activation. One major arm of this involves the catalytic subunit of the protein phosphatase 2 (PP2A) family of serine/threonine phosphatases, which mediates a wide variety of cellular dephosphorylation events [26]. The PP2A catalytic subunit directly associates with MST1/2, as well as MAP4K, dephosphorylating and inactivating core kinase cascade members [23,27]. Additionally, serine threonine kinase 25 (STK25), a STRIPAK-associated GCKIII kinase, can act to both inhibit and activate the Hippo kinase cascade respectively via direct phosphorylation of SAV1, thereby inhibiting SAV1’s inhibitory role within the STRIPAK’s complex [28], as well as via directly phosphorylating and activating LATS1/2 [29].

From a functional perspective, the kinase module generally acts in a tumour suppressive manner, phosphorylating and inactivating the transcriptional module members, YAP (Yorkie or Yki) and its paralogue TAZ (encoded for by *YAP1* and *WWTR1* genes respectively), which were identified as oncogenes shortly after their initial discovery [30,31]. Phosphorylated and inactive YAP/TAZ are retained in the cytoplasm of the cell and subsequently degraded. In the absence of an active kinase cascade, nuclear YAP/TAZ binds to the TEAD family of transcription factors, consisting of TEAD1–4 (Scalloped or Sd), which serve to activate the expression of pro-proliferative and survival enhancing gene programs [32–35]. Post-translational modifications are critical in the regulation of the Hippo pathway, with the core kinase cascade acting through sequential serine/threonine phosphorylation of its members to ultimately phosphorylate and inhibit YAP/TAZ on multiple residues. These include five core serine residues in YAP that when collectively mutated to phosphorylation-resistant alanine, result in a hyperactive YAP protein [36]. Phosphorylation at serine residue 127 has generally been most commonly studied in relation to YAP activity as relates to oncogenesis, partly due to the ability of this phosphorylation step to inhibit *in vitro* transformation [37], while it has also been found necessary for 14-3-3 protein binding [36], with 14-3-3 a known mediator of cytoplasmic retention [38]. Pragmatically, serine YAP 127 is likely also the most studied phosphorylation site due to well-functioning, commercially available site-specific antibodies. YAP is further regulated by a variety of tyrosine kinases, including Src-family kinases c-Src and YES1, which phosphorylate YAP at multiple positions, though predominantly Y357, driving YAP activation and transcription of anti-apoptotic [39] and regenerative [40] genes, as well as transformation [41], while inhibiting transcription of genes involved in differentiation [42]. Conversely, there is evidence to suggest that c-Abl-mediated phosphorylation of YAP at Y357 induces pro-apoptotic genes in response to DNA damage [43], suggesting a context-specific role of tyrosine phosphorylation in YAP activity related to tumorigenesis. Beyond regulation via phosphorylation, TEAD activity has recently been found to be dependent on nuclear/cytoplasmic translocation [44] as well as palmitoylation [45,46], a post-translational modification by which proteins are tagged by a fatty acid side chain. It has been suggested that TEAD has the capacity to palmitoylate itself [47], with this autopalmitoylation facilitating activity, as it functions to increase protein stability. The variety of kinase activators and activating markers observed across the pathway highlights the diversity in the signals regulating activity of the core Hippo pathway components.

## Hippo pathway dysregulation in cancer

### Genomic perturbation of kinase cascade

Although the Hippo signalling pathway is commonly dysregulated across a range of cancer types, mutations within the pathway are relatively rare: elements of the core kinase module are inactivated via mutation in common cancer types, though these are relatively infrequent events, typically present in <10% cancer cases [12,48]. However, the pathway’s direct ability to drive cancer onset and development are highlighted in both animal models, where YAP/TAZ hyperactivity drives onset of multiple types of cancer and metastasis, and in some rare varieties of human cancers, where loss-of-function mutations and deletions of kinase module genes are common (Table 1). In general, mutations within the Hippo pathway are somatic, potentially due to the importance of the pathway in regulating early development embryogenesis. A notable exception to this is *NF2*, which is mutated in the hereditary condition neurofibromatosis type 2, which causes development of benign schwannoma, meningiomas and ependymomas [49]. In the context of cancer, NF2 was consequently initially linked to oncogenesis through this association of *NF2* mutations and neurofibromatosis type 2 due to the observed generation of peripheral and central nervous system tumours [50]. Early research in mice suggested that truncation of just one copy of the mouse orthologue, *Nf2* [51], was sufficient to induce a spectrum of different cancer types, including osteosarcoma, lymphoma, lung adenocarcinoma and hepatocellular carcinoma [52]. Since these initial findings, *NF2* has been found to be loss-of-function mutated in ∼20–40% pleural mesothelioma (PM) cases [53–56] and 7% of renal cell carcinoma (RCC) patients [57], with a high degree of subtype-specificity noted, as exemplified by the papillary subtype of RCC, which exhibits a mutation frequency of 23% [58]. NF2 is also infrequently mutated in hepatocellular carcinoma (2%) and intrahepatic cholangiocarcinoma (5%) [59], highlighting NF2’s critical role as a tumour suppressor.

**Table 1:** Hippo pathway dysregulation and human cancers

Cancer type	Hippo component	Impact in cancer	Evidence
Breast cancer	YAP/TAZ	Nuclear translocation of TAZ is associated with the highly aggressive, triple-negative subtype of breast cancer [305]; YAP and TAZ facilitate stem cell-like properties in cancer cells [297,306]	Immunohistochemical assay of tumour tissue microarray; *in vitro* and *in vivo* experimentation
EHE	YAP/TAZ	Widespread fusions of TAZ, with recurrence through infrequent YAP fusions [98–100]	Whole-exome sequencing, cytogenetic analysis of patient samples
Ependymoma and Meningioma	YAP	Subpopulations of patients with YAP fusions [101,102,105]	Methylome characterisation and molecular inversion probe analysis of patient samples
Glioblastoma	YAP/TAZ	Transcriptional regulators of stem-like cell gene programs [145]	scRNA-seq of clinical samples
Hepatocellular carcinoma and Cholangiocarcinoma	YAP	YAP up-regulation leads to drug resistance *in vitro* [157] and worse prognosis in patients [154]; YAP activity shows contextual tumour suppressive ability, where overexpression in peritumoral hepatocytes leads to tumour clearance [215]	*In vitro* experiments and qPCR of clinical tissue; immunocompetent mice models
Mesothelioma	NF2, LATS1/2, SAV1	Frequently deleted/loss-of-function mutated in patients [12,54]	Whole-exome sequencing of patient samples
Non-small cell lung cancer (NSCLC)	YAP/TAZ	YAP enriched in nucleus in tumour relative to healthy tissue [307]; YAP and TAZ overexpression correlates to poor survival in NSCLC patients [308,309]; YAP and TAZ maintain stemness in NSCLC cancer cells [310]	Immunofluorescent staining of tumour tissue; immunohistochemcial quantification and RNA-seq; *in vitro* spheroid models of lung cancer
Osteosarcoma	YAP/TAZ	YAP protein levels up-regulated in OS cancer patients [311], with YAP/TAZ staining showing prognostic potential [312]; SOX2-Hippo axis activates YAP, maintaining cancer stem cell populations [148,251]	Immunohistochemistry of patient tumour microarray; *in vitro* analyses
Pancreatic ductal adenocarcinoma	YAP/TAZ	Associated with the highly aggressive, squamous subtype of PDAC [201,313]	RNA-seq and whole-exome sequencing of patient samples
Prostate cancer	YAP	Facilitates castration-resistant growth and invasiveness [314,315]; up-regulated in CRPC, but down-regulated in NEPC subtypes [210,316]; hydroxylation suppresses cell invasion [209]	*In vitro* and *in vivo* experimentation; clinical RNA-seq datasets; cell line analysis
Uveal melanoma	YAP	Gα_q/11_ mutant UM cells are dependent on YAP for oncogenic growth [178,317]	*In vitro* and *in vivo* experimentation

Non-exhaustive list of cancers in which the Hippo pathway is dysregulated, with a breakdown of the kind of perturbation observed, the pathway component(s) affected, and supporting evidence.

NF2 (or Merlin) is an upstream regulator of the core kinase cascade and is activated in response to a variety of cellular stresses, many of which are present during the process of oncogenesis and metastatic niche formation. For example, serum factors such as lysophosphatidic acid (LPA), a phospholipid historically associated with *in vitro* tumorigenesis [60,61], act as activators of YAP/TAZ. This is evidenced by LATS1/2 activation and subsequent YAP and TAZ inactivation on serum starvation in HEK293A and U2OS cells [62,63], a phenomenon mediated by NF2 [64]. In a similar fashion, NF2 also partially orchestrates the inactivation of YAP via phosphorylation observed on glucose starvation [64]. NF2 is a junctional protein and acts as a mediator of contact inhibition, the process by which motility and proliferation are impeded in regions of high cell density. In this capacity, NF2 is activated via a range of effectors, with NF2 historically shown to be essential for the formation of adherens junctions, the cell–cell complexes that facilitate adhesion and instigate contact inhibition [65]. Integrins can also activate an RAC/PAK1 axis, which in turn phosphorylates and inactivates NF2 (at S518), possibly through NF2’s function as a scaffold to bring LATS and YAP in close proximity [17], while cell–cell interactions inhibit YAP/TAZ-induced transcription via NF2’s regulation of FASN-mediated TEAD palmitoylation [46].

Tumours are known regions of poor nutrient availability due to the metabolic activation inherent to cancer [66], alongside reduced perfusion of metabolites and serum-derived factors [67], therefore the ability to escape an anti-proliferative response to glucose/serum-factor starvation would confer an advantage to NF2-mutant tumour initiators. The potential to proliferate and retain motility within a cell-dense region of tissue would also likely facilitate tumour growth and metastasis on loss of NF2. This, therefore, reinforces the likelihood that NF2 enacts its tumour suppressive role via the ability to act as a sensor of cellular stress and regulate a response to both serum/glucose starvation and contact inhibition.

### YAP/TAZ domain organisation and fusion events

YAP and TAZ consist of an N-terminal TEAD-binding domain (TBD), which wraps around the globular structure that defines TEAD proteins, interacting at three distinct interfaces [68,69]. Outside of cancer, mutations in TEAD are present in Sveinsson’s chorioretinal atrophy, an autosomal dominant eye disease, which prevents YAP/TAZ-TEAD binding [70–72]. Mutations in residues essential for YAP binding in TEADs were shown to disrupt transformation in MCF10A breast cancer cells and metastasis of a range of cellular model systems *in vivo* [69,73]. A 14-3-3 binding domain sits just downstream of the TBD; this includes the S127 residue, whose phosphorylation status determines 14-3-3 protein binding [74]. YAP and TAZ both contain WW domains, named for two highly evolutionarily conserved tryptophan residues contained within the domain [75]. Of note, there are a number of components within the Hippo signalling kinase cascade that contain PPxY, WW-binding motifs, including LATS1/2 and MST1/2 [76–78]. To explore the potential role of the WW domain in facilitating interactions between core cascade kinases and YAP/TAZ, a study focused on a fragment of the fly YAP orthologue, Yki, lacking a WW domain. Similar to the intact Yki, this fragment retains the ability to be phosphorylated by Wts, suggesting that this is independent of WW binding to Wts and regulation of Yki phosphorylation [79]. The WW domain in YAP is, however, essential for its activity in order to drive proliferation *in vitro* and *in vivo* [79,80] and can facilitate association between YAP and a variety of transcription factors, such as ERBB4 [81,82], JUNB [83] and RUNX2 [84], suggesting a TEAD-independent role for YAP as a transcriptional co-activator. A total of eight isoforms of YAP have been reported [85], with two major isoform types: YAP1 and YAP2, which contain one and two WW domains respectively [81]. These isoforms are in general functionally similar, however YAP2 has been shown to activate apoptosis in HEK293 cells [86], whereas YAP2L, a variant of YAP2, appears capable of dimerising to enhance oncogenic capacity [87]. The second WW domain associated with YAP2 may also have a functional role, as it has demonstrated potential as an enhancer of YAP’s potential as a transcriptional co-activator [81,88]. TAZ encoded by *WWTR1* is also expressed as several isoforms, some of which have markedly different functions [89,90], bringing additional complexities into the Hippo pathway. YAP and TAZ contain a relatively unstructured C-terminal transactivation domain (TAD) [91,92], which facilitate transformation *in vitro*, driving proliferation, migration and invasion [93,94], though is dispensable for anchorage-independent growth *in vitro* [93]. A PDZ-binding domain sits just downstream of the TAD, at the COOH terminus of YAP/TAZ, which is essential for nuclear translocation [95,96]. Evidence demonstrates that the TBD, TAD and PDZ-binding domains are all essential for YAP/TAZ function as a co-activator of TEAD-mediated transcription *in vitro* [88]. Finally, both YAP and TAZ contain relatively high levels of intrinsic disorder spanning across the entirety of the protein structures [97], suggesting a high degree of structural flexibility outside of short, highly conserved domains, may facilitate protein function.

**Figure 2:**
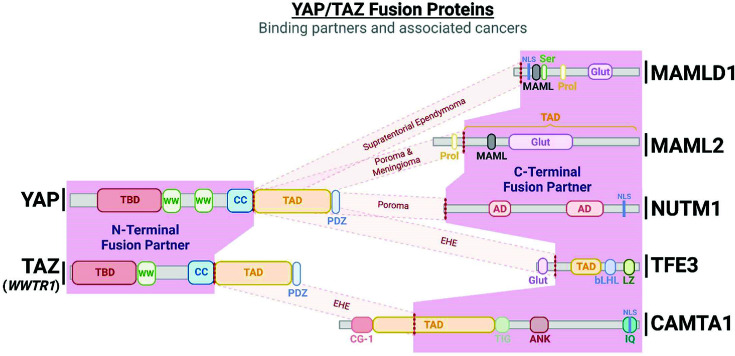
YAP/TAZ fusion partners and associated cancers Protein schematics showing the structures and domains of YAP/TAZ proteins (left) and common fusion partners in specific cancers (right). The location of frequent fusion breaks are denoted (red dashed lines), with common fusions and associated cancer types highlighted in red resulting in chimeric transcription factors. TAZ is encoded by *WWTR1*. Abbreviations: AD, acidic domain; ANK, ankyrin repeat region; bHLH, basic helix–loop–helix; CC, coiled-coil domain; CG-1, CG-1 DNA-binding domain; Glut, glutamine-rich region; IQ, IQ calmodulin-binding motif; LZ, leucine zipper; MAML, mastermind-like domain; PDZ, PDZ-binding domain; Prol, proline-rich region; ser, serine-rich region; TIG, transcription factor immunoglobulin domain.

Downstream of the kinase cascade, multiple activating fusions of YAP and TAZ, the transcriptional co-activators of the Hippo pathway, have been documented in a variety of rare cancer variants (Figure 2). An exemplar of this is epithelioid hemangioendothelioma (EHE), a vascular sarcoma that presents in a range of anatomical positions, thus exhibiting a variable prognosis, however with a tendency to metastasise early in its clinical course. This rare cancer-type is characterised by a near ubiquitous translocation resulting in a fusion protein of TAZ-CAMTA1 present in the vast majority (>90%) of patients [98], with an additional, less frequent YAP-TFE3 fusion protein also observed in a subpopulation of those diagnosed with EHE [99,100]. Another fusion example is present in a rare subtype of glioma, supratentorial ependymoma, in which a subset of ∼10% of patients exhibit a YAP-MAMLD1 or a highly infrequent YAP-FAM118B fusion [101,102]. The group of ependymomas with YAP1 fusions occurs predominantly in children [103]. In poroma (benign) and porocarcinoma (malignant) tumours, which arise from sweat glands, YAP-MAML2 and YAP-NUTM1 fusions are present in >80% and ∼60% of patients respectively [104,105]. Finally, YAP–MAML2 fusion is also observed in a subset of sporadic NF2 wildtype meningioma, whose methylomes mirror NF2 mutant tumours [106], and has also been reported in nasopharyngeal carcinoma [107].

In both EHE and ependymoma, YAP fusion events drive the transcription of TEAD target genes. To demonstrate this, a mouse model was developed expressing a tamoxifen-inducible knockin of TAZ-CAMTA1, which exhibits human-like EHE with a concurrent enrichment of YAP/TAZ target genes and pathways to a similar degree as in human disease [108]. Additionally, Hippo pathway dysregulation has been shown to result in tumorigenesis similar to that seen in cancer types involving YAP/TAZ fusion proteins, with both expression of constitutively active YAP or KO of LATS1/2 in a subpopulation of neuronal precursor cells in mice resulting in the formation of ependymoma-like tumours and up-regulation of YAP/TAZ target genes [109]. This core involvement of YAP/TAZ fusion proteins and target genes in the transformation of nervous tissue has been probed in more detail via preclinical experimentation, which has shown that cancer cells exhibiting a YAP-MAML2 fusion are dependent on the fusion protein, with loss associated with decreased viability, while also displaying increased expression of YAP/TAZ signature genes [110]. Experiments using HEK293 cells highlight that TADs of YAP-fusion partners are critical in driving the enhanced YAP activity associated with fusions and drive Hippo kinase cascade resistant nuclear localisation [111]. This suggests the substitution of the TAD and PDZ domains of YAP likely facilitates a constitutively nuclear, and hence active, YAP, which may account for the observation that these fusion proteins universally retain the N-terminal TEAD interacting domain, but not the C-terminal part of YAP and TAZ. The regularity of YAP fusion proteins in ependymomas and meningiomas, tumour types associated with neurofibromatosis 2, alongside preliminary findings to suggest exclusivity with NF2 mutation [101,106], is further evidence that Hippo pathway dysregulation is a common driver of oncogenesis in these rare cancer types of the nervous system.

Many of the rare cancer types that exhibit frequent YAP/TAZ fusions are associated with a generally low mutational burden, with supratentorial ependymomas characterised by fusion proteins in just two oncogenes (YAP and RELA) [101,103], while in EHE, <20% of patients exhibit a canonical oncogenic alteration beyond YAP/TAZ fusion [112]. This suggests that these chromosomal rearrangements are capable of transforming cells with relative genomic stability, further reinforcing the oncogenic potential of YAP/TAZ activity. In contrast with this, porocarcinomas generally display a higher degree of heterogeneity, more in line with most commonly studied cancer types [113], with frequent activation/inactivation of a range of oncogenic/tumour suppressive drivers [114]. However, it is apparent that fusion partners play a role in tumour development, with C-terminal fusion fragments exhibiting frequent conservation of nuclear localisation signal (NLS) and TAD motifs [98,115–118] (Figure 2), as demonstrated by glutamine-rich and highly acidic regions historically associated with TF-binding transactivation domains [119,120]. This is further evidenced by a case of porocarcinoma reported with a BRD3-NUTM1 fusion, independent of YAP rearrangement [121]. Collectively, these findings emphasise directly that YAP and TAZ, in combination with functional fragments of select fusion partners, are essential drivers in a subset of rare cancer varieties, likely in a highly context-specific manner, given the relative absence of YAP/TAZ fusions in cancer types arising from different tissues.

**Figure 3:**
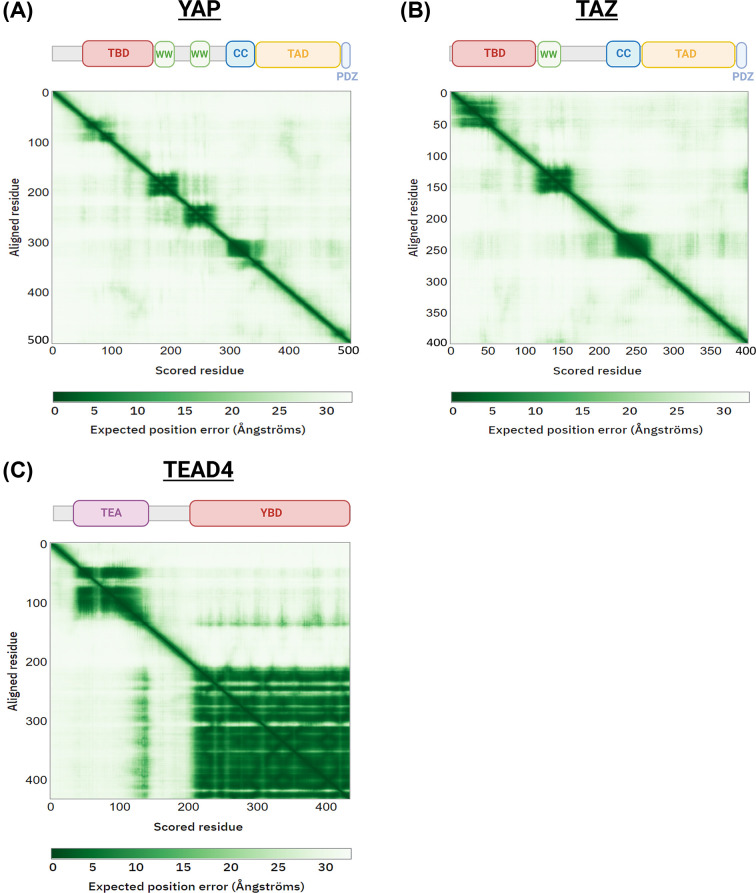
Intrinsic disorder of YAP and TAZ (**A**) Schematic of YAP protein structure, overlaid on to AlphaFold prediction expected position error of folded domains. Darker colours show a higher confidence in predicted relationship between residues. In general, a high level of predicted error persists throughout the various YAP domains, with just WW and CC domains exhibiting high levels of structural predictability. (**B**) Schematic of TAZ, as in (A), highlighting the high levels of intrinsic disorder that exists outside WW and CC domains. (**C**) Schematic of TEAD4, as in (A), with a higher degree of confidence in protein structure prediction observed throughout, as compared with YAP and TAZ proteins, suggesting a higher degree of structural order in TEAD4. Abbreviations: CC, coiled-coil domain; PDZ, PDZ-binding domain; TEA, TEA domain; YBD, YAP-binding domain.

### YAP/TAZ activation in cancer

Beyond genomic perturbation of the Hippo components, dysregulation at the transcriptional level is observed across a broad range of cancer types. Both *in vitro* and *in vivo* studies have reinforced YAP and TAZ’s role in driving proliferation in cancer cells and tumour tissue, as well as cellular migration, metastasis and resistance to therapeutics [122–124]. YAP drives transcription of integral cell cycle genes, such as the cell cycle transcription factor *FOXM1* and its target *CCND1* (encoding cyclin D1) in PM cells [32]. These pro-proliferative transcriptional programs may be driven in part by YAP/TAZ–TEAD association with the cell cycle transcription factor complex, AP-1. In a variety of cancer cell lines, YAP, TAZ and TEAD are found to colocalise with AP-1 to regulatory elements of the genome and are necessary for AP-1-mediated expression of cell cycle transcriptional programs, driving cancer cell proliferation and tumorigenesis in *in vivo* xenograft models [83,125,126]. Hyperactive YAP/TAZ also provides cells with a metabolic competitive advantage under nutrient-limiting conditions, in part by up-regulating glucose and high-affinity amino acid transporters [127–129]. This is especially relevant in the context of cancer development, where YAP/TAZ-regulated metabolic gene programs likely play critical roles in nutrient-poor environments such as the tumour microenvironment [130,131] and potentially facilitate the metabolic transformation to aerobic glycolysis historically associated with tumorigenesis [132–134]. There is also evidence that this metabolic IQ calmodulin-binding motif shift, often referred to as the ‘Warburg Effect’ [135], can act to drive YAP/TAZ activation, potentially instantiating a positive feedback loop within the cancer setting [136,137]. Additionally, there is accumulating evidence that YAP/TAZ activity is associated with pro-survival programs and the development of resistance to commonly used therapeutics. As an example, YAP mediates autophagy activation and survival in response to nutrient deprivation in breast cancer cells, likely due to YAP-TEAD-induced gene expression [34]. YAP mediates resistance to RAF and MEK inhibition in a range of cancer types, with a clear synergy between YAP knockdown (KD) and therapeutic inhibition of either BRAF with vemurafenib or MEK with trametinib [33]. Probing the molecular basis of the association between YAP/TAZ and pro-cancerous pathways is therefore a primary focus of recent cancer research.

As YAP/TAZ are key regulators of early developmental processes [138,139], it is not surprising that they frequently act in a pro-oncogenic capacity when not finely regulated, and drive the stemness and plasticity associated with cancer development, including metastasis, infiltration and resistance to therapeutics [140,141]. Epithelial-to-mesenchymal transition (EMT) is a critical step in most types of early oncogenesis, whereby terminally differentiated cells undergo a dedifferentiation, assuming stem-like properties. In Py2Ts, a murine breast cancer cell line proposed as a model to study EMT [142], YAP/TAZ–TEAD interactions are essential in driving EMT and expression of EMT transcriptional programs [143]. In the context of human cancers, YAP/TAZ in some instances act downstream of MEKK3 (or MAP3K3) to maintain stemness in pancreatic cancer cells [144], while collation of single cell RNA-seq (scRNA-seq) datasets from patients with glioblastoma (GBM) revealed that YAP and TAZ drive a regulatory network associated with the GBM stem cell state [145]. YAP/TAZ may in part be driven by a SOX2-Hippo axis; SOX2 is a master transcription factor historically associated with pluripotency and stem cell maintenance [146] and is also often linked to tumorigenesis [147]. This SOX2-Hippo axis has been shown to directly inhibit NF2 and activate YAP, driving cell plasticity in both osteosarcoma and GBM cell lines [148]. YAP/TAZ activity plays a prominent role in early liver development, with multiple studies in zebrafish and mouse showing that repression of the Hippo kinase cascade/hyperactivation of YAP is sufficient to cause overgrowth within the liver and biliary duct [149–153]. YAP is also overexpressed in cholangiocarcinoma, cancer of the biliary duct, with increased expression corresponding with a worse patient outcome [154], in some rare instances due to loss of NF2 function [155,156], while YAP promotes therapeutic resistance in *in vitro* models of hepatocellular carcinoma [157]. This serves to highlight the requirement within cells to finely balance YAP/TAZ activity to promote healthy organogenesis during development and avoid tumorigenesis post-development.

While a range of transcriptional programs have been proposed to act as drivers of oncogenesis downstream of the transcriptional module of the Hippo pathway, it is important to understand how these transcriptional effects are mediated by YAP/TAZ–TEAD while considering potential therapeutic approaches to inhibit these processes. In an NF2 mutated breast cancer cell line model, YAP, TAZ and TEAD were shown to preferentially localise to enhancer elements [125]. These non-coding segments of the genome are typically just several hundred base pairs in length and act in a *cis*-regulatory manner to modulate transcription via the recruitment of transcription factors and transcriptional co-activators [158]. The term super-enhancer was originally conceived to describe key regulators of stem cell transcription programmes in the context of embryonic stem cells [159–161] and are defined as elongated clusters of enhancer regions with increased transcription factor densities, which exhibit an enhanced capacity to drive transcription of select genes [159]. Given their association with embryonic stem cells and the master regulators that drive cellular plasticity, many genes regulated by super-enhancers encode proteins that are essential for development and pluripotency, such as SOX2, Nanog and OCT4 [159,162]. These can be hijacked in cancer cells to promote tumorigenesis, with breast and colorectal cancer cells found to exhibit increased levels of the H3K27Ac mark of epigenetic activation at super-enhancer sites of known oncogenes, such as *c-Myc* and *ESR1* (encoding oestrogen receptor α (ERα)), relative to non-malignant tissue as determined by ChIP-seq [163]. Core Hippo pathway components associate closely with these super-enhancers, with YAP and TAZ populating super-enhancer regions as a result of their association with BRD4, a member of the bromodomain and extraterminal domain (BET) family of epigenetic readers [164]. This interaction mediates the transcription of pro-proliferative genes and is required for cancer cell viability [164]. A model for transcription factor and binding partner capture within super-enhancers has been proposed [165], by which liquid–liquid phase separation (LLPS), whereby a homogenous mixture demixes forming separate condensed and dilute phases, drives the formation of membraneless organelles [166]. This might be of particular relevance to YAP and TAZ, which readily undergo LLPS under specific stimuli, such as upon osmotic challenges, as a result of intrinsic disorder, a phenomenon that drives widespread transcriptional effects within cells [157,167]. For example, YAP forms condensates under conditions of osmotic stress, driving nuclear localisation and transforming chromatin topology, resulting in a clustering of accessible chromatin and the transcription of YAP/TAZ target genes [168]. In contrast with this, TAZ undergoes LLPS at steady-state *in vitro*, suggesting that phase separation is particularly important for TAZ relative to YAP [97]. This LLPS is inhibited by Hippo kinase cascade activation, while TAZ condensates colocalise with TEADs and markers of super-enhancers such as BRD4 and MED1, activating expression of YAP/TAZ target genes [97]. As further evidence, in mice embryonic stem cells, hyperactive Yap induced by Mst1/2 KO is found to phase-separate and colocalise with master transcriptional regulators Sox2, Nanog and Oct4, disrupting stem cell differentiation [169] and highlighting the importance of LLPS in coordinating association between YAP/TAZ and super-enhancer elements.

### The role of G protein-coupled receptor signalling in regulating the Hippo pathway in cancer

G protein-coupled receptors (GPCRs) are transmembrane proteins capable of binding a diverse set of ligands [170], facilitating the response to a range of extracellular stimuli and inducing various signalling cascades via the activation of G proteins. G proteins function in signal transduction as GTPases, acting via the hydrolysis of GTP to GDP, and comprise two major families: monomeric small GTPases [171] and heterotrimeric G proteins that consist of α, β and γ subunits. Within the heterotrimeric G protein family, the Gα subunit is the primary functional element required for GDP/GTP binding and decides G protein nomenclature [172], with multiple subfamilies of G protein α-subunits including Gα_12/13_, Gα_q/11_ and Gα_s_. GPCRs activate G proteins by inducing exchange of GDP for GTP, a process mediated by guanine nucleotide exchange factors [173]. Both GPCRs and G proteins are frequently mutated across a vast variety of cancer types, with GPCR, Gα_q/11_ and Gα_s_ mutations present in ∼20, 4 and 6% of all human cancers [174], while recent work has suggested that G protein disruption may be even more common than previously observed when mutually exclusive mutations across G protein families are considered [175]. GPCRs and coupled G proteins differentially regulate the core kinases of the Hippo signalling pathway, with YAP found to be activated by serum constituents such as the bioactive signalling lipids LPA and sphingosine 1-phosphate (S1P), but inhibited by metabolic stress hormones [62]. These effects are mediated by the kinases of the Hippo pathway, with LATS1/2 inhibited by Gα_12/13_ and Gα_q/11_ subfamilies and activated by the Gα_s_ subfamily of heterotrimeric G proteins [62]. To explore the role of G proteins in regulating YAP/TAZ in the context of cancer, the role of activating Gα_q/11_ mutations, which occur frequently in uveal melanoma (UM) [176,177], in driving YAP-mediated oncogenic processes was examined in UM cell lines. UM cells with mutations in *GNAQ* or *GNA11*, which encode Gα_q/11_ proteins, demonstrate increased YAP activity, while ectopic expression of mutant Gα_q/11_ also induce YAP activation in HEK293 cells [178]. This is of particular relevance, given that Gα_q/11_ mutations enhanced susceptibility to YAP-TEAD inhibition via treatment with the YAP-TEAD inhibitor verteporfin *in vitro* [178], suggesting that cancers driven by these G protein subfamilies, as well as GPCRs that regulate them, may be vulnerable to therapeutic targeting of Hippo pathway effectors.

Downstream of heterotrimeric G proteins, small GTPases, in particular RhoA, a member of the Ras superfamily Rho GTPases, are also known to regulate Hippo pathway activity. The family of LPA-activated GPCRs activate Rho-dependent signalling in response to LPA and absence of mechanical force [179,180], an axis which, when active, inhibits LATS1/2 [62]. This phenomenon, instigated by Gα_12/13_, utilises the upstream mechanosensory component of the Hippo pathway [2], with activated RhoA driving F-actin assembly and leading to LATS1/2 inactivation [181]. Recently, super-resolution dSTORM imaging further resolved this process, highlighting how YAP activity is inhibited in response to cell contact and mechanotransduction via RhoA repression [182]. These observations point to the importance of RhoA as a mediator of GPCR regulation of Hippo signalling, which is of particular relevance in cancer given RhoA plays a key role in transformation induced by aberrant GPCR signalling [183]. Across a range of cancer types, *RHOA* overexpression is associated with oncogenesis, in patients and cell line models, as well as advanced disease [184–187], further highlighting its role in progression of cancer. The involvement of RhoA in oncogenesis may be in part due to its ability to restructure the actin cytoskeleton and drive motility, as oncogene-mediated RhoA activation induces cancer cell migration and invasion [188,189]. However, recent *in vitro* and *in vivo* experiments have shown that YAP further mediates the oncogenic potential of RhoA, driving the expression of downstream transcriptional programs that induce restructuring of the cytoskeleton and extracellular matrix, enhancing cancer cell invasion [190,191], while LPA-mediated RhoA activation and subsequent dephosphorylation of YAP induce migration in ovarian cancer cells [192].

The oncogenic Kaposi sarcoma-associated herpesvirus (KSAH), a virus responsible for the initiation of Kaposi sarcoma, generally in immunocompromised patients such as those with an advanced HIV infection, depends on a viral GPCR (vGPCR) element to induce tumorigenesis [193]. Tumorigenesis induced by vGPCR is mediated by and dependent on the Hippo pathway *in vitro*, with vGPCR inhibiting LATS1/2 through G_12/13_, Gα_q/11_ and RhoA, leading to increased activation of YAP/TAZ and enhanced proliferation and migration [194]. It is also worth noting that there are a number of members of the Ras subfamily of GTPases which are frequently mutated in human cancers, including HRAS, NRAS and most commonly, KRAS, with activating Ras mutations found in ∼20% of cancer patients [195,196]. YAP/TAZ facilitate tumorigenesis in Ras-driven cancers [188,189], possibly via regulation of overlapping, downstream transcriptional targets [197], and can act as a surrogate for oncogenic Ras *in vitro* when KRAS is suppressed in cell lines from a range of cancer types [198]. This is particularly relevant in pancreatic ductal adenocarcinoma (PDAC), in which the aggressive squamous subtype exhibit independence of oncogenic KRAS [199], a near constitutive driver of PDAC [200]. *YAP1* expression levels are associated with poor patient outcome and the squamous subtype in PDAC [201,202], with YAP acting to bypass KRAS dependency in pancreatic cancer cell lines [201], suggesting an ability to induce transcription of targets up-regulated on aberrant KRAS signalling. Collectively, this work reinforces the role of YAP/TAZ as important effectors of oncogenic GPCR, G protein and general GTPase signalling.

### YAP/TAZ as tumour suppressors

Despite the clear role YAP/TAZ play in tumorigenesis, some studies have linked their activity to anti-cancer pathways in a variety of cancer types. For example, low levels of *YAP1* expression have been associated with a significantly poorer prognosis in haematological cancers, with *in vitro*-based research highlighting the role YAP plays in reducing proliferation in multiple myeloma cells [203], likely mediated via interaction between YAP and the pro-apoptotic p73 [204]. Similarly, in small cell lung cancers (SCLCs), *YAP1* is minimally expressed or even absent from most cases, particularly those of neuroendocrine lineage, with just a subpopulation of patients displaying high levels of expression [205]. Loss of heterozygosity in chromosome 11q22-q23, the region containing *YAP1*, has historically been observed in breast cancer [206], while YAP knockout or KD in a variety of breast cancer cell lines yields a reduction in tumorigenic potential, as determined by capacity for anchorage-independent growth, migration, and ability to form xenografts in mice [207,208]. A recent study highlights that cell lines originating from multiple tissues of origin exhibited an increase in *in vitro* metastatic potential after KD of *YAP1*, further suggesting a tumour suppressive role; however, this suppressive phenotype was only observed when YAP was hydroxylated in a prostate cancer cell line [209]. These observations are surprising, given that YAP has typically been found to drive cell proliferation and oncogenesis in prostate cancer [141]. There is however a subset of neuroendocrine prostate cancers exhibiting a silencing of YAP [210], suggesting that YAP/TAZ may exhibit a context-dependent tumour suppressive function via post-translational modification. Importantly, a range of these studies exclusively focus on YAP, and therefore TAZ compensatory roles [211,212] might not be picked up. Consequently, both TAZ and YAP function and activity are critical to evaluate in order to obtain firm conclusions.

Recently, a transcriptional profile for cancers that exhibit YAP silencing showed a binary switch from YAP dependency to independency apparent across pan-cancer datasets. The present study revealed that YAP and TAZ act as tumour suppressors selectively in retinoblastomas and SCLCs exhibiting loss of the *RB1* gene, which is mutated in the vast majority of both cancer types [205,213]. Probing cell line and clinical transcriptomes revealed the existence of multiple clusters of cancers; a relatively small subpopulation of cancer types in which YAP/TAZ are silenced, constituting haematological malignancies and small cell neuroendocrine cancers, and the majority of those in which YAP/TAZ are actively expressed, primarily consisting of solid, non-neuroendocrine tumours [214]. To compound the idea of YAP/TAZ acting as tumour suppressors or oncogenes in this binary fashion, a recent study leveraged mice models of cholangiocarcinoma and hepatocellular carcinoma to study the impact of YAP/TAZ dysregulation in peritumoral immune cells. This showed that hyperactivation of YAP in tumour cells promotes cancer progression, while in surrounding hepatocytes, YAP/TAZ hyperactivity leads to tumour suppression [215]. These results highlight the nuanced and contextual role the Hippo pathway plays in tumour development, although a majority of cancers clearly exhibit some dependence on YAP/TAZ transcriptional activation.

## Therapeutics and Hippo signalling

### Direct targeting of YAP/TAZ-TEAD

Early attempts to target YAP/TAZ activity focused on disrupting YAP/TAZ-TEAD binding, with the first compound to efficiently inhibit this interaction discovered by using a YAP-TEAD luciferase reporter assay. This approach identified verteporfin, a Food and Drug Administration (FDA)-approved member of the porphyrin family historically used as a photosensitiser to treat macular degeneration [216], to also act as an inhibitor of YAP–TEAD transcription, which selectively inhibited tumour growth in murine models of YAP-driven hepatocellular carcinoma [217]. However, the clinical potential of verteporfin in targeting YAP-TEAD driven cancers is limited as off-target effects have been reported, the cytotoxicity associated with verteporfin treatment having been shown as acting independently of YAP inhibition in a range of cancer cell models [218,219]. This has led to more recent efforts to develop allosteric inhibitors that disrupt the YAP–TEAD interaction, with a variety of compounds having been shown to exhibit potential [220–222] (Table 2), though future studies are required to validate specificity before clinical efficacy can be considered.

**Table 2:** Inhibitors of YAP/TAZ activity

Compound name	Mechanism of action	Clinical viability
Verteporfin	Disrupts YAP–TEAD association [217], possibly partly via cytoplasmic sequestration of YAP [318]	Approved for clinical use and historically used as non-cancer therapeutic [216]; however, YAP/TAZ independent anti-cancer potential and cell death reported *in vitro* [218,219]
CA3	Reduces expression of *YAP1*, as well as YAP–TEAD transcriptional activity [319]	No clinical data, however anti-cancer potential is validated *in vivo* [320,321]
Cyclic YAP-like peptides	Acts as a competitive inhibitor of intact YAP, disrupting YAP–TEAD interaction [322]	Peptides are non-cell permeable and therefore require additional intracellular delivery tools before being used clinically [322]
Super-TDU	Mimics the structure of the TDU domain of VGLL4, found to competitively bind TEAD, acting to disrupt the YAP–TEAD interaction [323]	No clinical data; however, a variety of similar acting compounds have recently been patented [324] and are viable for testing
Flufenamic acid	Binds the central, hydrophobic pocket of TEADs, disrupting YAP–TEAD transcriptional activity; however, YAP–TEAD binding is maintained [325]	Approved for clinical use as non-steroidal anti-inflammatory drug (NSAID) [326], however no clinical data on anti-cancer potential
TED-347	Flufenamic acid derived molecule that binds TEAD palmitate pocket, displacing YAP and inhibiting YAP–TEAD transcriptional activity [221]	No clinical data, though likely similar pharmacological profile to flufenamic acid
Various palmitoylation inhibitors	A selection of small molecule inhibitors have been recently identified that bind the palmitoylation pocket of TEAD, acting as a dominant-negative inhibitor of YAP/TAZ activity [223–225]	No clinical use data, as compounds are in early stages of development/testing, with clinical trials currently recruiting (NCT04665206)

List of therapeutics developed to target the transcriptional module of the Hippo pathway, with corresponding mechanism of action and potential to reposition clinically.

As previously discussed, TEAD activity is dependent on palmitoylation [45–47], a phenomenon that can be targeted molecularly. To this end, a variety of TEAD inhibitors have been developed that target palmitoylation sites conserved across TEAD isoforms, inducing a dominant-negative effect on transcriptional regulation [47,223]. The anti-cancer potential of this therapeutic approach has been validated in a mesothelioma xenograft model, in which NF2-deficient cancer cells exhibited sensitivity to inhibition of TEAD palmitoylation [224]. While these broad-acting TEAD inhibitors are currently under development with the intention to reposition to clinical testing in the near future, isoform-specific inhibitors are also being considered as potential tools for research. This is exemplified by the design of a selective inhibitor of TEAD3 [225], whose function is relatively unknown in the context of cancer progression, relative to the other TEAD isoforms [226–228]. Additionally, MST1/2 inhibitors have been developed, with the intention to utilise these to therapeutically stimulate tissue repair and regeneration [229]. However, as a subset of cancers exist in which YAP/TAZ act as putative tumour suppressors [205,210,214], there is a possibility that these cancers may be vulnerable to inhibition of the core Hippo kinase cascade; this approach would however require validation and caution, as MST1/2 classically act as tumour suppressors [230–232].

Strikingly, there is a distinct lack of inhibitors that target YAP or TAZ directly; to evaluate why this is the case, it is necessary to consider protein structure. YAP and TAZ are intrinsically disordered, suggesting a high degree of structural flexibility [97]. Recent advances in deep learning have facilitated *in silico* protein prediction such that current modelling approaches have demonstrated accurate prediction of protein structure to near experimental quality [233]. Implementing this methodology, AlphaFold predictions validate the extent of disorder inherent to YAP and TAZ (Figure 3), with just WW and CC domains predicted with high confidence, along with a small subsection of the TEAD-binding domain. This acts as an indicator that there may be few regions within YAP and TAZ that are susceptible to therapeutic inhibition, as development of small molecule inhibitors will be limited to those select structures within the proteins with a high degree of order. There is also difficulties inherent to inhibition of YAP/TAZ activity via targeting upstream regulatory elements, as this would necessitate the therapeutic activation of the Hippo kinase module. Historically, kinase inhibitors represent a major subfamily of anti-cancer compounds, with a variety of therapeutic avenues involving kinase inhibition showing clinical efficacy in the context of cancer [234,235]. As the majority of kinases involved in cancer act in a pro-oncogenic capacity, there are limited therapeutic options available in terms of activators of kinases, meaning novel compounds would need to be developed to this end. However, there are some clinically viable kinase activators available, as exemplified by compounds known to activate AMP-activated kinase (AMPK), an important sensor of metabolic stress in cells [236]. A variety of therapeutics, including metformin, a drug widely used to treat diabetes [237], induce activation of AMPK [238], some of which have shown anti-cancer potential in preclinical models of various cancer types [239–241], acting as proof that a therapeutic option to switch on the core kinase cascade of the Hippo pathway is viable. Approaches targeting YAP/TAZ protein stability, mRNA levels and translation are alternative approaches that might be productive.

### Indirect targeting of YAP/TAZ and associated pathways

Beyond directly targeting the components within the Hippo pathway, an alternative approach may be to indirectly inhibit Hippo pathway effectors via upstream regulators. An attractive example of this may be to inhibit GPCRs associated with G_12/13_ and G_q/11_ subfamilies of G proteins, which activate YAP/TAZ [62,178]. Many drugs commonly used for the treatment of a variety of diseases and conditions interact with GPCRs or proteins associated with GPCRs, with 35% of compounds listed as approved by the United States FDA targeting GPCRs [242]. However, very few of these are utilised specifically within the context of cancer as anti-tumorigenic agents [243], with recent work serving to highlight the potential in GPCR inhibitor discovery and repositioning in cancer [244]. Additionally, G_q/11_ activating mutations have been found to modulate YAP/TAZ activity via focal adhesion kinase (FAK), highlighting potential in inhibition of FAK as a cancer therapy, with *in vitro* validation in UM cell lines [245]. This therapeutic approach is validated experimentally, as NF2 expression levels predict efficacy of FAK inhibition in cells derived from pancreatic cancer patients [246]. Additionally, an improved response is observed with *NF2* KD *in vitro* and *in vivo* [246], suggesting that YAP/TAZ activity likely positively correlates with sensitivity to FAK inhibition, given NF2’s function as an activator of the Hippo kinase cascade (Figure 1).

An alternative approach is to target the super-enhancer elements that coordinate regulation of expression directly with YAP/TAZ to inhibit the activation of transcriptional programmes involved in tumorigenesis. This approach appears effective in preclinical experiments, with BRD repression via treatment with JQ1, a broad-acting BET inhibitor [247], and *BRD2/3/4* KD showing anti-cancer potential *in vivo* models of YAP/TAZ-addicted breast cancer [164]. This treatment strategy has additionally been validated across a range of cancer types [248,249], reinforcing its potential as a therapeutic for the treatment of cancer. Additionally, a mechanism by which the stemness of osteosarcoma cells dependent on the SOX2-YAP axis can be exploited to therapeutically induce adipogenesis has been proposed. In this manner, stem-like tumour cells can be treated with thiazolidinediones, which function as agonists of PPARγ, a key transcription factor and nutrient sensor which drives adipogenesis when activated [250]. Adipogenic differentiation in stem cell-like cancer cells stimulated by PPARγ activation in this manner has been shown to limit tumorigenicity *in vitro* and *in vivo* [251]. Targeting transcriptional targets of YAP/TAZ–TEAD is yet an additional approach; however, since YAP/TAZ regulates hundreds of genes [12,164,252], this is challenging, but might be a feasible context-dependent complimentary strategy.

In liver and prostate cancer mice cell models, hyperactive YAP recruits macrophages [253] and myeloid-derived suppressor cells (MDSCs) [254] respectively, in both cases acting with TEAD to initiate the expression of immunosuppressive cytokines such as CXCL5, CXCL1/2 and CCL2, repressing the immune response. Mouse models of PDAC have further validated this observation, showing that Yap deletion inhibits MDSC recruitment and polarisation, likely via inhibition of Yap-Tead target gene expression [255], a phenomenon that leads to T-cell reactivation and tissue regeneration [255]. Both YAP and TAZ can also drive the expression of programmed cell death 1 ligand (PDL1) [256,257], which binds to and activates PD1, an immune checkpoint receptor that acts to suppress the immune response [258,259]. These observations are relevant in the context of tumour initiation, as immunosuppression is often employed by cancer cells throughout tumorigenesis to evade and survive the immune response, while there has been a concerted effort throughout the past decade to position checkpoint inhibitors to combat this in a clinical setting [260]. This clear role of YAP/TAZ-TEAD in driving the tumour cell intrinsic signals necessary for the oncogenic immunosuppressive phenotype suggests the potential in targeting YAP/TAZ in combination with checkpoint inhibition [13], an experimental approach validated preclinically [261]. This discovery is of particular relevance currently given the recent focus on leveraging immunotherapy to manage a wide variety of cancer types [262,263], with a combinatorial approach that simultaneously targets YAP/TAZ activity potentially overcoming the resistance associated with immunotherapy [264,265]. Noteworthily, enforced expression of constitutively activated YAP/TAZ in a range of tissue culture and cancer cells regularly induces expression of inflammatory cytokines [83,266–270], which directly highlights that the Hippo pathway is likely a cellular nexus that links epithelial, fibroblasts and endothelial inflammatory responses and proliferation during cancer onset and development [13,261,266].

In contrast, LATS1/2 knockout across multiple types of cancer cells in xenograft studies stimulates the anti-cancer immune response via release of nucleic acid-rich extracellular vesicles [271–273]. LATS1/2 loss in these studies [271] enhances tumour immunogenicity, which promotes anti-tumor immune responses and tumour regression leading to a reduction in tumorigenicity *in* *vivo* [271], suggesting that the Hippo pathway plays a complex role in the involvement of the immune response to oncogenesis. Overall, the context-dependent role the Hippo pathway plays in immune oncology warrants further examination in order to uncover the interplay and complexities between Hippo pathway components and the immune system [274].

### Future targeting of Hippo signalling in cancer

Given the relatively recent emergence of the Hippo pathway’s role as a cancer driver, much has been learned as to how YAP/TAZ regulates cancer initiation and downstream oncogenic processes, as well as how they are in turn regulated by the upstream, generally tumour suppressive kinase module. This progress has given rise to a concerted effort to develop therapeutics that target components within the Hippo signalling pathway [140,275,276], as highlighted by the recent arrival of a variety of promising TEAD inhibitors, one of which [224] is currently undergoing testing in an actively recruiting clinical trial involving patients with PM (NCT04665206). These therapeutics are of particular importance given the widespread association of YAP/TAZ activity with cancer progression and the preclinical evidence supporting the potential of inhibition of YAP/TAZ-TEAD driven expression in suppressing cancer growth. Beyond considering single-agent treatment with a YAP/TAZ/TEAD inhibitor, positioning such therapeutics alongside standard-of-care treatments may present an optimal choice, given the role of downstream Hippo pathway signalling in driving resistance to a variety of anti-cancer drugs [265]. There is additional potential in targeting cancer cells dependent on YAP/TAZ by indirectly targeting the Hippo pathway, disrupting regulation of upstream or downstream components such as GPCR or super-enhancer components, respectively [277–279]. A variety of such strategies involving the targeting of YAP/TAZ activity have been described in the past, including the leveraging of approved, clinically established drugs such as the AMPK agonist metformin and the statin family of HMG-CoA reductase (HMGCR) inhibitors, classically used to treat hypercholesterolaemia. These compounds disrupt YAP/TAZ activity via metformin-induced activation of AMPK [238] and subsequent direct and indirect phosphorylation of YAP [280,281], as well as the disruption of the mevalonate pathway by statins [282], which results in inhibited geranylgeranylation of RhoA and its displacement from the cell membrane, leading to LATS1/2- and MST1/2-independent YAP phosphorylation [283,284]. Recent retrospective analyses of clinical trials have highlighted the anti-cancer potential of these therapeutics [285,286], though these findings should be taken with caution as prospective trials are necessary to robustly confirm findings [287]. Despite the promise of targeting YAP/TAZ addiction across a broad range of cancer types, there exists the potential that tumour cells may switch from YAP/TAZ dependency to escape therapeutic sensitivity. This is reinforced by the observation that prostate adenocarcinoma cells lose YAP/TAZ expression as they transition to the more aggressive neuroendocrine subtype [214]. These considerations must therefore be taken into account when testing clinical efficacy of next-generation inhibitors.

From a prognostic perspective, the detection of high levels of YAP/TAZ transcriptional activity is generally a uniform indicator of reduced overall survival in patients across cohorts of multiple cancer types, further highlighting the need for therapeutics that target YAP/TAZ addiction in cancer. To exemplify this association between enhanced YAP/TAZ activity and cancer prognosis, a significant decrease in survival time is observed in patients across a variety of The Cancer Genome Atlas (TCGA) cohorts that exhibit an above median average expression of *bona fide* downstream targets of YAP/TAZ [12]. High levels of nuclear YAP also correlate with poor clinical outcomes in a variety of cancers [122,123] and is observed in 70% of PM patients [288]. However, determining YAP/TAZ activity is not always a simple task, partially due to the complex nature of Hippo pathway regulation. There are a variety of players involved in activating and inactivating the core kinase cascade that constitutes the pathway, many of which are complicit in cancer development. For example, EGFR, which is frequently mutated to a constitutively active form in lung and breast cancer [289,290], phosphorylates and represses MOB1, inactivating LATS1/2 and resulting in hyperactive YAP/TAZ [291].

**Figure 4:**
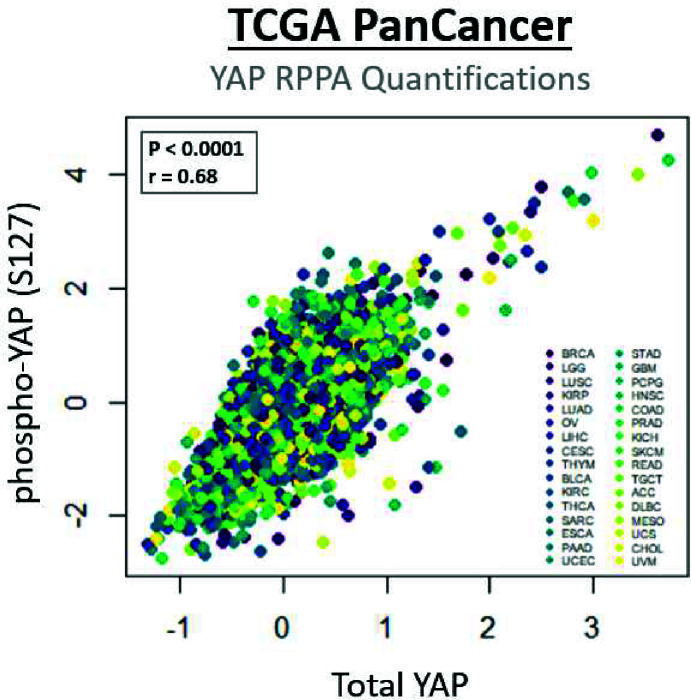
Implementing YAP levels as prognostic indicator of YAP activity Scatter-plot showing correlation between total YAP levels and levels of pYAP (S127) in patients across a range of cancer types. A strong and significant positive correlation exists between the levels of the two proteins, indicating that in patients with high levels of pYAP (S127), a concurrent increase in total YAP levels is observed. Points shown comprise RPPA data across the pan-cancer dataset (obtained from the Genomic Data Comms portal; https://gdc.cancer.gov/), normalised across cancer subtypes (level 4). Correlation coefficients and *P*-values were determined via Spearman method.

Beyond the complex interplay between canonical cancer drivers and the Hippo pathway, further difficulties lie in determining a single prognostic indicator for YAP/TAZ activity from patient biopsies. Conventionally, expression of YAP/TAZ at the transcriptional and protein levels have been interpreted as a metric for activity, which fails to fully account for the nuance in regulation of YAP/TAZ at the post-transcriptional and post-translational levels. As an example of this, quantification of levels of YAP phosphorylated at serine residue 127 (pYAP (S127)) are frequently utilised as a measure of Hippo kinase cascade activity, both in preclinical and clinical cancer samples [292–294]; however, within TCGA reverse-phase protein array (RPPA) datasets, which show quantification of protein levels within patient samples, there is a striking positive correlation between levels of pYAP (S127) and total YAP (Figure 4). This suggests that in patient samples with high levels of YAP phosphorylation at S127, there may be a pool of compensatory, non-phosphorylated and active YAP, indicating that quantification of phospho-YAP alone may be insufficient to determine activity. Another limitation in many studies is the over-reliance on S127 phosphorylation as a sole marker of YAP activity. When initially described in the context of cancer, phosphorylation at S381 was also found to inhibit *in vitro* transformation [37], while cyclin-dependent kinase 1 (CDK1), a key driver of mitosis, phosphorylates YAP at three alternative residues, positively regulating oncogenesis *in vitro* [295]. Collectively, this suggests the need for a biologically meaningful metric of YAP/TAZ activity that fully accounts for the various mechanisms that regulate activity, with quantification of *bona fide* downstream targets perhaps representing an ideal approach [12].

There are many outstanding questions as to the function of the Hippo pathway in the context of cancer. For example, the extent of the functional distinction between YAP and TAZ, the two primary downstream effectors of the pathway. Most research discussed throughout displays a clear focus on YAP, which has been functionally characterised to a far greater extent than TAZ. However, this general focus on YAP is not necessarily reflective of the importance each component plays in signalling. There is evidence to suggest that both YAP and TAZ are regulated similarly in response to a variety of stresses across a range of cellular *in vitro* models such as HEK293A cells [296], suggesting some degree of functional redundancy, while YAP disruption has a greater effect on transcription as compared with TAZ [125]. In contrast, the degree of evolutionary conservation of TAZ within vertebrates [14] suggests some potential functional divergence, while research has validated a non-redundancy in functionality between YAP and TAZ [91]. Within the context of cancer, TAZ plays an important role independent of YAP, particularly in driving cancer stem cell properties, as has been observed in a variety of *in vitro* cancer cell models [297–299]. TAZ, in some instances acts as an upstream effector of the master transcriptional regulator of stemness, SOX2 [300], in contrast with YAP which is directly regulated by SOX2 [148]. This deviation in functionality means some caution must be exercised when interpreting *in vitro* experiments that focus solely on YAP; for example, where YAP’s role as a tumour suppressor in cancer cells has been observed and validated via KD, it is possible that TAZ may have acted to compensate for loss of YAP via hyperactivation or increase expression, a phenomenon that has been observed *in vitro* and *in vivo* in the past [296,301,302].

Multiple types and levels of cellular regulatory feedback are prominent features centring on Hippo signalling, tightly and dynamically regulating this potent signalling pathway [3,15]. It is possible that, if parts of these integrations are offset due to mutations or epigenetic silencing in prominent signalling nodes outside the core Hippo pathway machinery, or mechanochemical alterations within the cellular niche, that this might impose unregulated hyperactive YAP/TAZ, causing cancer. There is additionally evidence that various upstream components of the Hippo pathway can act independently of the downstream transcriptional module, with NF2 exhibiting pleiotropy in mesothelioma [303] and LATS1/2 acting as a regulator of ERα stability in breast cancer cells independent of kinase activity [304]. The upstream regulatory kinase cascade (Figure 1) also undoubtedly have a range of additional substrates that likely feedback and integrate into the Hippo pathway [1,3]. Collectively, the findings discussed herein highlight the need to develop our understanding of this complex signalling pathway as it relates to cancer progression and development, elucidate further the upstream regulatory elements, and disentangle the nuanced context-dependent ability of components to act as tumour suppressors and oncogenes. In so doing, we can more effectively consider stratifying patients according to Hippo pathway dysregulation and develop therapeutic options for clinical management of YAP/TAZ-driven cancers.

## Open Access

Open access for this article was enabled by the participation of The University of Edinburgh in an all-inclusive *Read & Publish* pilot with Portland Press and the Biochemical Society under a transformative agreement with *JISC*.

## Abbreviations

AMPK: AMP-activated kinase
BET: bromodomain and extraterminal domain
CDK1: cyclin dependent kinase 1
EHE: epithelioid hemangioendothelioma
EMT: epithelial-to-mesenchymal transition
ERα: oestrogen receptor α
FAK: focal adhesion kinase
FDA: Food and Drug Administration
GBM: glioblastoma
GPCR: G protein-coupled receptor
HMGCR: HMG-CoA Reductase
KD: knockdown
LLPS: liquid–liquid phase separation
LPA: lysophosphatidic acid
MAP4K: mitogen-activated kinase kinase kinase kinase
MDSC: myeloid-derived suppressor cell
NLS: nuclear localisation signal
PDAC: pancreatic ductal adenocarcinoma
PM: pleural mesothelioma
PP2A: protein phosphatase 2
RCC: renal cell carcinoma
SCLC: small cell lung cancer
STK25: serine threonine kinase 25
STRIPAK: striatin-interacting phosphatase and kinase
TAD: transactivation domain
TBD: TEAD-binding domain
TCGA: The Cancer Genome Atlas
UM: uveal melanoma
vGPCR: viral GPCR

## References

[1] Rausch V. and Hansen C.G. (2020) The Hippo pathway, YAP/TAZ, and the plasma membrane. Trends Cell Biol. 30, 32–48 10.1016/j.tcb.2019.10.005 31806419

[2] Dupont S., Morsut L., Aragona M., Enzo E., Giulitti S., Cordenonsi M. et al. (2011) Role of YAP/TAZ in mechanotransduction. Nature 474, 179–183 10.1038/nature10137 21654799

[3] Park J. and Hansen C.G. (2021) Cellular feedback dynamics and multilevel regulation driven by the hippo pathway. Biochem. Soc. Trans. 49, 1515–1527 10.1042/BST20200253 34374419 PMC8421037

[4] Justice R.W., Zilian O., Woods D.F., Noll M. and Bryant P.J. (1995) The Drosophila tumor suppressor gene warts encodes a homolog of human myotonic dystrophy kinase and is required for the control of cell shape and proliferation. Genes Dev. 9, 534–546 10.1101/gad.9.5.534 7698644

[5] Xu T., Wang W., Zhang S., Stewart R.A. and Yu W. (1995) Identifying tumor suppressors in genetic mosaics: the Drosophila lats gene encodes a putative protein kinase. Development 121, 1053–1063 10.1242/dev.121.4.1053 7743921

[6] Wu S., Huang J., Dong J. and Pan D. (2003) Hippo encodes a Ste-20 family protein kinase that restricts cell proliferation and promotes apoptosis in conjunction with salvador and warts. Cell 114, 445–456 10.1016/S0092-8674(03)00549-X 12941273

[7] Udan R.S., Kango-Singh M., Nolo R., Tao C. and Halder G. (2003) Hippo promotes proliferation arrest and apoptosis in the Salvador/Warts pathway. Nat. Cell Biol. 5, 914–920 10.1038/ncb1050 14502294

[8] Nishioka N., Inoue K., Adachi K., Kiyonari H., Ota M., Ralston A. et al. (2009) The Hippo signaling pathway components Lats and Yap pattern Tead4 activity to distinguish mouse trophectoderm from inner cell mass. Dev. Cell 16, 398–410 10.1016/j.devcel.2009.02.003 19289085

[9] Morin-Kensicki E.M., Boone B.N., Howell M., Stonebraker J.R., Teed J., Alb J.G. et al. (2006) Defects in yolk sac vasculogenesis, chorioallantoic fusion, and embryonic axis elongation in mice with targeted disruption of Yap65. Mol. Cell. Biol. 26, 77–87 10.1128/MCB.26.1.77-87.2006 16354681 PMC1317614

[10] Harvey K.F., Pfleger C.M. and Hariharan I.K. (2003) The Drosophila Mst ortholog, hippo, restricts growth and cell proliferation and promotes apoptosis. Cell 114, 457–467 10.1016/S0092-8674(03)00557-9 12941274

[11] Moroishi T., Hansen C.G. and Guan K.-L. (2015) The emerging roles of YAP and TAZ in cancer. Nat. Rev. Cancer 15, 73–79 10.1038/nrc3876 25592648 PMC4562315

[12] Wang Y., Xu X., Maglic D., Dill M.T., Mojumdar K., Ng P.K.-S. et al. (2018) Comprehensive molecular characterization of the Hippo signaling pathway in cancer. Cell Rep. 25, 1304.e5–1317.e5 10.1016/j.celrep.2018.10.001 30380420 PMC6326181

[13] Dey A., Varelas X. and Guan K.-L. (2020) Targeting the Hippo pathway in cancer, fibrosis, wound healing and regenerative medicine. Nat. Rev. Drug Discov. 19, 480–494 10.1038/s41573-020-0070-z 32555376 PMC7880238

[14] Hilman D. and Gat U. (2011) The evolutionary history of YAP and the Hippo/YAP pathway. Mol. Biol. Evol. 28, 2403–2417 10.1093/molbev/msr065 21415026

[15] Hansen C.G., Moroishi T. and Guan K.-L. (2015) YAP and TAZ: a nexus for Hippo signaling and beyond. Trends Cell Biol. 25, 499–513 10.1016/j.tcb.2015.05.002 26045258 PMC4554827

[16] Wei X., Shimizu T. and Lai Z.-C. (2007) Mob as tumor suppressor is activated by Hippo kinase for growth inhibition in Drosophila. EMBO J. 26, 1772–1781 10.1038/sj.emboj.7601630 17347649 PMC1847660

[17] Sabra H., Brunner M., Mandati V., Wehrle-Haller B., Lallemand D., Ribba A.-S. et al. (2017) β1 integrin-dependent Rac/group I PAK signaling mediates YAP activation of Yes-associated protein 1 (YAP1) via NF2/merlin. J. Biol. Chem. 292, 19179–19197 10.1074/jbc.M117.808063 28972170 PMC5702661

[18] Yin F., Yu J., Zheng Y., Chen Q., Zhang N. and Pan D. (2013) Spatial organization of Hippo signaling at the plasma membrane mediated by the tumor suppressor Merlin/NF2. Cell 154, 1342–1355 10.1016/j.cell.2013.08.025 24012335 PMC3835333

[19] Genevet A., Wehr M.C., Brain R., Thompson B.J. and Tapon N. (2010) Kibra is a regulator of the Salvador/Warts/Hippo signaling network. Dev. Cell 18, 300–308 10.1016/j.devcel.2009.12.011 20159599 PMC2845807

[20] Braicu C., Buse M., Busuioc C., Drula R., Gulei D., Raduly L. et al. (2019) A comprehensive review on MAPK: a promising therapeutic target in cancer. Cancers (Basel) 11, 1618 10.3390/cancers11101618 PMC6827047 31652660

[21] Meng Z., Moroishi T., Mottier-Pavie V., Plouffe S.W., Hansen C.G., Hong A.W. et al. (2015) MAP4K family kinases act in parallel to MST1/2 to activate LATS1/2 in the Hippo pathway. Nat. Commun. 6, 8357 10.1038/ncomms9357 26437443 PMC4600732

[22] Seo G., Han H., Vargas R.E., Yang B., Li X. and Wang W. (2020) MAP4K interactome reveals STRN4 as a key STRIPAK complex component in Hippo pathway regulation. Cell Rep. 32, 107860 10.1016/j.celrep.2020.107860 32640226 PMC7382313

[23] Chen R., Xie R., Meng Z., Ma S. and Guan K.-L. (2019) STRIPAK integrates upstream signals to initiate the Hippo kinase cascade. Nat. Cell Biol. 21, 1565–1577 10.1038/s41556-019-0426-y 31792377

[24] Shi Z., Jiao S. and Zhou Z. (2016) STRIPAK complexes in cell signaling and cancer. Oncogene 35, 4549–4557 10.1038/onc.2016.9 26876214

[25] Ribeiro P.S., Josué F., Wepf A., Wehr M.C., Rinner O., Kelly G. et al. (2010) Combined functional genomic and proteomic approaches identify a PP2A complex as a negative regulator of Hippo signaling. Mol. Cell 39, 521–534 10.1016/j.molcel.2010.08.002 20797625

[26] Jong C.J., Merrill R.A., Wilkerson E.M., Herring L.E., Graves L.M. and Strack S. (2020) Reduction of protein phosphatase 2A (PP2A) complexity reveals cellular functions and dephosphorylation motifs of the PP2A/B’δ holoenzyme. J. Biol. Chem. 295, 5654–5668 10.1074/jbc.RA119.011270 32156701 PMC7186168

[27] Tang Y., Fang G., Guo F., Zhang H., Chen X., An L. et al. (2020) Selective inhibition of STRN3-containing PP2A phosphatase restores Hippo tumor-suppressor activity in gastric cancer. Cancer Cell 38, 115.e9–128.e9 10.1016/j.ccell.2020.05.019 32589942

[28] Bae S.J., Ni L. and Luo X. (2020) STK25 suppresses Hippo signaling by regulating SAV1-STRIPAK antagonism. eLife 9, e54863 10.7554/eLife.54863 32292165 PMC7182433

[29] Lim S., Hermance N., Mudianto T., Mustaly H.M., Mauricio I.P.M., Vittoria M.A. et al. (2019) Identification of the kinase STK25 as an upstream activator of LATS signaling. Nat. Commun. 10, 1547 10.1038/s41467-019-09597-w 30948712 PMC6449379

[30] Overholtzer M., Zhang J., Smolen G.A., Muir B., Li W., Sgroi D.C. et al. (2006) Transforming properties of YAP, a candidate oncogene on the chromosome 11q22 amplicon. Proc. Natl. Acad. Sci. U.S.A. 103, 12405–12410 10.1073/pnas.0605579103 16894141 PMC1533802

[31] Steinhardt A.A., Gayyed M.F., Klein A.P., Dong J., Maitra A., Pan D. et al. (2008) Expression of Yes-associated protein in common solid tumors. Hum. Pathol. 39, 1582–1589 10.1016/j.humpath.2008.04.012 18703216 PMC2720436

[32] Mizuno T., Murakami H., Fujii M., Ishiguro F., Tanaka I., Kondo Y. et al. (2012) YAP induces malignant mesothelioma cell proliferation by upregulating transcription of cell cycle-promoting genes. Oncogene 31, 5117–5122 10.1038/onc.2012.5 22286761

[33] Lin L., Sabnis A.J., Chan E., Olivas V., Cade L., Pazarentzos E. et al. (2015) The Hippo effector YAP promotes resistance to RAF- and MEK-targeted cancer therapies. Nat. Genet. 47, 250–256 10.1038/ng.3218 25665005 PMC4930244

[34] Song Q., Mao B., Cheng J., Gao Y., Jiang K., Chen J. et al. (2015) YAP enhances autophagic flux to promote breast cancer cell survival in response to nutrient deprivation. PLoS ONE 10, e0120790 10.1371/journal.pone.0120790 25811979 PMC4374846

[35] Koo J.H., Plouffe S.W., Meng Z., Lee D.-H., Yang D., Lim D.-S. et al. (2020) Induction of AP-1 by YAP/TAZ contributes to cell proliferation and organ growth. Genes Dev. 34, 72–86 10.1101/gad.331546.119 31831627 PMC6938666

[36] Zhao B., Wei X., Li W., Udan R.S., Yang Q., Kim J. et al. (2007) Inactivation of YAP oncoprotein by the Hippo pathway is involved in cell contact inhibition and tissue growth control. Genes Dev. 21, 2747–2761 10.1101/gad.1602907 17974916 PMC2045129

[37] Zhao B., Li L., Tumaneng K., Wang C.-Y. and Guan K.-L. (2010) A coordinated phosphorylation by Lats and CK1 regulates YAP stability through SCF(beta-TRCP). Genes Dev. 24, 72–85 10.1101/gad.1843810 20048001 PMC2802193

[38] Muslin A.J. and Xing H. (2000) 14-3-3 proteins: regulation of subcellular localization by molecular interference. Cell. Signal. 12, 703–709 10.1016/S0898-6568(00)00131-5 11152955

[39] Rosenbluh J., Nijhawan D., Cox A.G., Li X., Neal J.T., Schafer E.J. et al. (2012) β-Catenin-driven cancers require a YAP1 transcriptional complex for survival and tumorigenesis. Cell 151, 1457–1473 10.1016/j.cell.2012.11.026 23245941 PMC3530160

[40] Taniguchi K., Wu L.-W., Grivennikov S.I., de Jong P.R., Lian I., Yu F.-X. et al. (2015) A gp130-Src-YAP module links inflammation to epithelial regeneration. Nature 519, 57–62 10.1038/nature14228 25731159 PMC4447318

[41] Li P., Silvis M.R., Honaker Y., Lien W.-H., Arron S.T. and Vasioukhin V. (2016) αE-catenin inhibits a Src-YAP1 oncogenic module that couples tyrosine kinases and the effector of Hippo signaling pathway. Genes Dev. 30, 798–811 10.1101/gad.274951.115 27013234 PMC4826396

[42] Zaidi S.K., Sullivan A.J., Medina R., Ito Y., van Wijnen A.J., Stein J.L. et al. (2004) Tyrosine phosphorylation controls Runx2-mediated subnuclear targeting of YAP to repress transcription. EMBO J. 23, 790–799 10.1038/sj.emboj.7600073 14765127 PMC380991

[43] Levy D., Adamovich Y., Reuven N. and Shaul Y. (2008) Yap1 phosphorylation by c-Abl is a critical step in selective activation of proapoptotic genes in response to DNA damage. Mol. Cell 29, 350–361 10.1016/j.molcel.2007.12.022 18280240

[44] Lin K.C., Moroishi T., Meng Z., Jeong H.-S., Plouffe S.W., Sekido Y. et al. (2017) Regulation of Hippo pathway transcription factor TEAD by p38 MAPK-induced cytoplasmic translocation. Nat. Cell Biol. 19, 996–1002 10.1038/ncb3581 28752853 PMC5541894

[45] Chan P., Han X., Zheng B., DeRan M., Yu J., Jarugumilli G.K. et al. (2016) Autopalmitoylation of TEAD proteins regulates transcriptional output of the Hippo pathway. Nat. Chem. Biol. 12, 282–289 10.1038/nchembio.2036 26900866 PMC4798901

[46] Kim N.-G. and Gumbiner B.M. (2019) Cell contact and Nf2/Merlin-dependent regulation of TEAD palmitoylation and activity. Proc. Natl. Acad. Sci. U.S.A. 116, 9877–9882 10.1073/pnas.1819400116 31043565 PMC6525549

[47] Noland C.L., Gierke S., Schnier P.D., Murray J., Sandoval W.N., Sagolla M. et al. (2016) Palmitoylation of TEAD transcription factors is required for their stability and function in Hippo pathway signaling. Structure 24, 179–186 10.1016/j.str.2015.11.005 26724994

[48] Yoo N.J., Park S.W. and Lee S.H. (2012) Mutational analysis of tumour suppressor gene NF2 in common solid cancers and acute leukaemias. Pathology 44, 29–32 10.1097/PAT.0b013e32834c3599 22081132

[49] Evans D.G.R. (2009) Neurofibromatosis 2 [Bilateral acoustic neurofibromatosis, central neurofibromatosis, NF2, neurofibromatosis type II]. Genet. Med. 11, 599–610 10.1097/GIM.0b013e3181ac9a27 19652604

[50] Begnami M.D., Palau M., Rushing E.J., Santi M. and Quezado M. (2007) Evaluation of NF2 gene deletion in sporadic schwannomas, meningiomas, and ependymomas by chromogenic in situ hybridization. Hum. Pathol. 38, 1345–1350 10.1016/j.humpath.2007.01.027 17509660 PMC2094208

[51] McClatchey A.I., Saotome I., Ramesh V., Gusella J.F. and Jacks T. (1997) The Nf2 tumor suppressor gene product is essential for extraembryonic development immediately prior to gastrulation. Genes Dev. 11, 1253–1265 10.1101/gad.11.10.1253 9171370

[52] McClatchey A.I., Saotome I., Mercer K., Crowley D., Gusella J.F., Bronson R.T. et al. (1998) Mice heterozygous for a mutation at the Nf2 tumor suppressor locus develop a range of highly metastatic tumors. Genes Dev. 12, 1121–1133 10.1101/gad.12.8.1121 9553042 PMC316711

[53] Bueno R., Stawiski E.W., Goldstein L.D., Durinck S., De Rienzo A., Modrusan Z. et al. (2016) Comprehensive genomic analysis of malignant pleural mesothelioma identifies recurrent mutations, gene fusions and splicing alterations. Nat. Genet. 48, 407–416 10.1038/ng.3520 26928227

[54] Hmeljak J., Sanchez-Vega F., Hoadley K.A., Shih J., Stewart C., Heiman D. et al. (2018) Integrative molecular characterization of malignant pleural mesothelioma. Cancer Discov. 8, 1548–1565 10.1158/2159-8290.CD-18-0804 30322867 PMC6310008

[55] Bianchi A.B., Mitsunaga S.I., Cheng J.Q., Klein W.M., Jhanwar S.C., Seizinger B. et al. (1995) High frequency of inactivating mutations in the neurofibromatosis type 2 gene (NF2) in primary malignant mesotheliomas. Proc. Natl. Acad. Sci. U.S.A. 92, 10854–10858 10.1073/pnas.92.24.10854 7479897 PMC40529

[56] Sekido Y., Pass H.I., Bader S., Mew D.J., Christman M.F., Gazdar A.F. et al. (1995) Neurofibromatosis type 2 (NF2) gene is somatically mutated in mesothelioma but not in lung cancer. Cancer Res. 55, 1227–1231 7882313

[57] Yakirevich E., Perrino C., Necchi A., Grivas P., Bratslavsky G., Shapiro O. et al. (2020) NF2 mutation-driven renal cell carcinomas (RCC): a comprehensive genomic profiling (CGP) study. J. Clin. Oncol. 38, 726–726 10.1200/JCO.2020.38.6_suppl.726

[58] Sourbier C., Liao P.-J., Ricketts C.J., Wei D., Yang Y., Baranes S.M. et al. (2018) Targeting loss of the Hippo signaling pathway in NF2 -deficient papillary kidney cancers. Oncotarget 9, 10723–10733 10.18632/oncotarget.24112 29535838 PMC5828210

[59] Zhang N., Zhao Z., Long J., Li H., Zhang B., Chen G. et al. (2017) Molecular alterations of the NF2 gene in hepatocellular carcinoma and intrahepatic cholangiocarcinoma. Oncol. Rep. 38, 3650–3658 10.3892/or.2017.6055 29130106

[60] Imamura F., Horai T., Mukai M., Shinkai K., Sawada M. and Akedo H. (1993) Induction of in vitro tumor cell invasion of cellular monolayers by lysophosphatidic acid or phospholipase D. Biochem. Biophys. Res. Commun. 193, 497–503 10.1006/bbrc.1993.1651 8390242

[61] Fishman D.A., Liu Y., Ellerbroek S.M. and Stack M.S. (2001) Lysophosphatidic acid promotes matrix metalloproteinase (MMP) activation and MMP-dependent invasion in ovarian cancer cells. Cancer Res. 61, 3194–3199 11306508

[62] Yu F.-X., Zhao B., Panupinthu N., Jewell J.L., Lian I., Wang L.H. et al. (2012) Regulation of the Hippo-YAP pathway by G-protein-coupled receptor signaling. Cell 150, 780–791 10.1016/j.cell.2012.06.037 22863277 PMC3433174

[63] Miller E., Yang J., DeRan M., Wu C., Su A.I., Bonamy G.M.C. et al. (2012) Identification of serum-derived sphingosine-1-phosphate as a small molecule regulator of YAP. Chem. Biol. 19, 955–962 10.1016/j.chembiol.2012.07.005 22884261

[64] Plouffe S.W., Meng Z., Lin K.C., Lin B., Hong A.W., Chun J.V. et al. (2016) Characterization of Hippo pathway components by gene inactivation. Mol. Cell 64, 993–1008 10.1016/j.molcel.2016.10.034 27912098 PMC5137798

[65] Lallemand D., Curto M., Saotome I., Giovannini M. and McClatchey A.I. (2003) NF2 deficiency promotes tumorigenesis and metastasis by destabilizing adherens junctions. Genes Dev. 17, 1090–1100 10.1101/gad.1054603 12695331 PMC196046

[66] Finicle B.T., Jayashankar V. and Edinger A.L. (2018) Nutrient scavenging in cancer. Nat. Rev. Cancer 18, 619–633 10.1038/s41568-018-0048-x 30097614

[67] Munir R., Lisec J., Swinnen J.V. and Zaidi N. (2019) Lipid metabolism in cancer cells under metabolic stress. Br. J. Cancer 120, 1090–1098 10.1038/s41416-019-0451-4 31092908 PMC6738079

[68] Li Z., Zhao B., Wang P., Chen F., Dong Z., Yang H. et al. (2010) Structural insights into the YAP and TEAD complex. Genes Dev. 24, 235–240 10.1101/gad.1865810 20123905 PMC2811825

[69] Chen L., Chan S.W., Zhang X., Walsh M., Lim C.J., Hong W. et al. (2010) Structural basis of YAP recognition by TEAD4 in the Hippo pathway. Genes Dev. 24, 290–300 10.1101/gad.1865310 20123908 PMC2811830

[70] Fossdal R., Jonasson F., Kristjansdottir G.T., Kong A., Stefansson H., Gosh S. et al. (2004) A novel TEAD1 mutation is the causative allele in Sveinsson’s chorioretinal atrophy (helicoid peripapillary chorioretinal degeneration). Hum. Mol. Genet. 13, 975–981 10.1093/hmg/ddh106 15016762

[71] Kitagawa M. (2007) A Sveinsson’s chorioretinal atrophy-associated missense mutation in mouse Tead1 affects its interaction with the co-factors YAP and TAZ. Biochem. Biophys. Res. Commun. 361, 1022–1026 10.1016/j.bbrc.2007.07.129 17689488

[72] Bokhovchuk F., Mesrouze Y., Izaac A., Meyerhofer M., Zimmermann C., Fontana P. et al. (2019) Molecular and structural characterization of a TEAD mutation at the origin of Sveinsson’s chorioretinal atrophy. FEBS J. 286, 2381–2398 10.1111/febs.14817 30903741

[73] Lamar J.M., Stern P., Liu H., Schindler J.W., Jiang Z.-G. and Hynes R.O. (2012) The Hippo pathway target, YAP, promotes metastasis through its TEAD-interaction domain. Proc. Natl. Acad. Sci. U.S.A. 109, E2441–E2450 10.1073/pnas.1212021109 22891335 PMC3443162

[74] Zhao B., Wei X., Li W., Udan R.S., Yang Q., Kim J. et al. (2007) Inactivation of YAP oncoprotein by the Hippo pathway is involved in cell contact inhibition and tissue growth control. Genes Dev. 21, 2747–2761 10.1101/gad.1602907 17974916 PMC2045129

[75] Bork P. and Sudol M. (1994) The WW domain: a signalling site in dystrophin? Trends Biochem. Sci. 19, 531–533 10.1016/0968-0004(94)90053-1 7846762

[76] Salah Z. and Aqeilan R.I. (2011) WW domain interactions regulate the Hippo tumor suppressor pathway. Cell Death Dis. 2, e172 10.1038/cddis.2011.53 21677687 PMC3168995

[77] Sudol M. and Harvey K.F. (2010) Modularity in the Hippo signaling pathway. Trends Biochem. Sci. 35, 627–633 10.1016/j.tibs.2010.05.010 20598891

[78] Furth N. and Aylon Y. (2017) The LATS1 and LATS2 tumor suppressors: beyond the Hippo pathway. Cell Death Differ. 24, 1488–1501 10.1038/cdd.2017.99 28644436 PMC5563998

[79] Oh H. and Irvine K.D. (2009) In vivo analysis of Yorkie phosphorylation sites. Oncogene 28, 1916–1927 10.1038/onc.2009.43 19330023 PMC2701235

[80] Zhao B., Kim J., Ye X., Lai Z.-C. and Guan K.-L. (2009) Both TEAD-binding and WW domains are required for the growth stimulation and oncogenic transformation activity of Yes-associated protein. Cancer Res. 69, 1089–1098 10.1158/0008-5472.CAN-08-2997 19141641

[81] Komuro A., Nagai M., Navin N.E. and Sudol M. (2003) WW domain-containing protein YAP associates with ErbB-4 and acts as a co-transcriptional activator for the carboxyl-terminal fragment of ErbB-4 that translocates to the nucleus. J. Biol. Chem. 278, 33334–33341 10.1074/jbc.M305597200 12807903

[82] Haskins J.W., Nguyen D.X. and Stern D.F. (2014) Neuregulin 1-activated ERBB4 interacts with YAP to induce Hippo pathway target genes and promote cell migration. Sci. Signal. 7, ra116 10.1126/scisignal.2005770 25492965 PMC4648367

[83] He L., Pratt H., Gao M., Wei F., Weng Z. and Struhl K. (2021) YAP and TAZ are transcriptional co-activators of AP-1 proteins and STAT3 during breast cellular transformation. eLife 10, e67312 10.7554/eLife.67312 34463254 PMC8463077

[84] Yagi R., Chen L.-F., Shigesada K., Murakami Y. and Ito Y. (1999) A WW domain-containing Yes-associated protein (YAP) is a novel transcriptional co-activator. EMBO J. 18, 2551–2562 10.1093/emboj/18.9.2551 10228168 PMC1171336

[85] Gaffney C.J., Oka T., Mazack V., Hilman D., Gat U., Muramatsu T. et al. (2012) Identification, basic characterization and evolutionary analysis of differentially spliced mRNA isoforms of human YAP1 gene. Gene 509, 215–222 10.1016/j.gene.2012.08.025 22939869 PMC3455135

[86] Oka T., Mazack V. and Sudol M. (2008) Mst2 and Lats kinases regulate apoptotic function of Yes kinase-associated protein (YAP). J. Biol. Chem. 283, 27534–27546 10.1074/jbc.M804380200 18640976

[87] Khanal P., Jia Z. and Yang X. (2018) Cysteine residues are essential for dimerization of Hippo pathway components YAP2L and TAZ. Sci. Rep. 8, 3485 10.1038/s41598-018-21828-6 29472569 PMC5823869

[88] Finch-Edmondson M.L., Strauss R.P., Clayton J.S., Yeoh G.C. and Callus B.A. (2016) Splice variant insertions in the C-terminus impairs YAP's transactivation domain. Biochem. Biophys. Rep. 6, 24–31 10.1016/j.bbrep.2016.02.015 28018981 PMC5176130

[89] Fang C., Li J., Qi S., Lei Y., Zeng Y., Yu P. et al. (2019) An alternatively transcribed TAZ variant negatively regulates JAK ‐ STAT signaling. EMBO Rep. 20, e47227 10.15252/embr.201847227 30979708 PMC6549033

[90] Callus B.A., Finch-Edmondson M.L., Fletcher S. and Wilton S.D. (2019) YAPping about and not forgetting TAZ. FEBS Lett. 593, 253–276 10.1002/1873-3468.13318 30570758

[91] Reggiani F., Gobbi G., Ciarrocchi A. and Sancisi V. (2021) YAP and TAZ are not identical twins. Trends Biochem. Sci. 46, 154–168 10.1016/j.tibs.2020.08.012 32981815

[92] Manning S.A., Kroeger B. and Harvey K.F. (2020) The regulation of Yorkie, YAP and TAZ: new insights into the Hippo pathway. Development 147, dev179069 10.1242/dev.179069 32341025

[93] Zhang X., Grusche F.A. and Harvey K.F. (2012) Control of tissue growth and cell transformation by the Salvador/Warts/Hippo pathway. PLoS ONE 7, e31994 10.1371/journal.pone.0031994 22359650 PMC3281119

[94] Xia Y., Chang T., Wang Y., Liu Y., Li W., Li M. et al. (2014) YAP promotes ovarian cancer cell tumorigenesis and is indicative of a poor prognosis for ovarian cancer patients. PLoS ONE 9, e91770 10.1371/journal.pone.0091770 24622501 PMC3951505

[95] Oka T. and Sudol M. (2009) Nuclear localization and pro-apoptotic signaling of YAP2 require intact PDZ-binding motif. Genes Cells 14, 607–615 10.1111/j.1365-2443.2009.01292.x 19371381

[96] Oka T., Remue E., Meerschaert K., Vanloo B., Boucherie C., Gfeller D. et al. (2010) Functional complexes between YAP2 and ZO-2 are PDZ domain-dependent, and regulate YAP2 nuclear localization and signalling1. Biochem. J. 432, 461–478 10.1042/BJ20100870 20868367

[97] Lu Y., Wu T., Gutman O., Lu H., Zhou Q., Henis Y.I. et al. (2020) Phase separation of TAZ compartmentalizes the transcription machinery to promote gene expression. Nat. Cell Biol. 22, 453–464 10.1038/s41556-020-0485-0 32203417 PMC11044910

[98] Tanas M.R., Sboner A., Oliveira A.M., Erickson-Johnson M.R., Hespelt J., Hanwright P.J. et al. (2011) Identification of a disease-defining gene fusion in epithelioid hemangioendothelioma. Sci. Transl. Med. 3, 98ra82 10.1126/scitranslmed.3002409 21885404

[99] Antonescu C.R., Le Loarer F., Mosquera J.-M., Sboner A., Zhang L., Chen C.-L. et al. (2013) Novel YAP1-TFE3 fusion defines a distinct subset of epithelioid hemangioendothelioma. Genes Chromosomes Cancer 52, 775–784 10.1002/gcc.22073 23737213 PMC4089994

[100] Lee S.J., Yang W.I., Chung W.-S. and Kim S.K. (2016) Epithelioid hemangioendotheliomas with TFE3 gene translocations are compossible with CAMTA1 gene rearrangements. Oncotarget 7, 7480–7488 10.18632/oncotarget.7060 26840265 PMC4884933

[101] Pajtler K.W., Witt H., Sill M., Jones D.T.W., Hovestadt V., Kratochwil F. et al. (2015) Molecular classification of ependymal tumors across all CNS compartments, histopathological grades, and age groups. Cancer Cell 27, 728–743 10.1016/j.ccell.2015.04.002 25965575 PMC4712639

[102] Andreiuolo F., Varlet P., Tauziède-Espariat A., Jünger S.T., Dörner E., Dreschmann V. et al. (2019) Childhood supratentorial ependymomas with YAP1-MAMLD1 fusion: an entity with characteristic clinical, radiological, cytogenetic and histopathological features. Brain Pathol. 29, 205–216 10.1111/bpa.12659 30246434 PMC7379249

[103] Pajtler K.W., Mack S.C., Ramaswamy V., Smith C.A., Witt H., Smith A. et al. (2017) The current consensus on the clinical management of intracranial ependymoma and its distinct molecular variants. Acta Neuropathol. 133, 5–12 10.1007/s00401-016-1643-0 27858204 PMC5209402

[104] Sekine S., Kiyono T., Ryo E., Ogawa R., Wakai S., Ichikawa H. et al. (2019) Recurrent YAP1-MAML2 and YAP1-NUTM1 fusions in poroma and porocarcinoma. J. Clin. Invest. 129, 3827–3832 10.1172/JCI126185 31145701 PMC6715383

[105] Russell‐Goldman E., Hornick J.L. and Hanna J. (2021) Utility of YAP1 and NUT immunohistochemistry in the diagnosis of porocarcinoma. J. Cutan. Pathol. 48, 403–410 10.1111/cup.13924 33222286

[106] Sievers P., Chiang J., Schrimpf D., Stichel D., Paramasivam N., Sill M. et al. (2020) YAP1-fusions in pediatric NF2-wildtype meningioma. Acta Neuropathol. 139, 215–218 10.1007/s00401-019-02095-9 31734728

[107] Valouev A., Weng Z., Sweeney R.T., Varma S., Le Q.-T., Kong C. et al. (2014) Discovery of recurrent structural variants in nasopharyngeal carcinoma. Genome Res. 24, 300–309 10.1101/gr.156224.113 24214394 PMC3912420

[108] Seavey C.N., Pobbati A.V., Hallett A., Ma S., Reynolds J.P., Kanai R. et al. (2021) WWTR1 (TAZ)-CAMTA1 gene fusion is sufficient to dysregulate YAP/TAZ signaling and drive epithelioid hemangioendothelioma tumorigenesis. Genes Dev. 35, 512–527 10.1101/gad.348220.120 33766982 PMC8015722

[109] Eder N., Roncaroli F., Domart M.-C., Horswell S., Andreiuolo F., Flynn H.R. et al. (2020) YAP1/TAZ drives ependymoma-like tumour formation in mice. Nat. Commun. 11, 2380 10.1038/s41467-020-16167-y 32404936 PMC7220953

[110] Picco G., Chen E.D., Alonso L.G., Behan F.M., Gonçalves E., Bignell G. et al. (2019) Functional linkage of gene fusions to cancer cell fitness assessed by pharmacological and CRISPR-Cas9 screening. Nat. Commun. 10, 2198 10.1038/s41467-019-09940-1 31097696 PMC6522557

[111] Szulzewsky F., Arora S., Hoellerbauer P., King C., Nathan E., Chan M. et al. (2020) Comparison of tumor-associated YAP1 fusions identifies a recurrent set of functions critical for oncogenesis. Genes Dev. 34, 1051–1064 10.1101/gad.338681.120 32675324 PMC7397849

[112] Rosenbaum E., Jadeja B., Xu B., Zhang L., Agaram N.P., Travis W. et al. (2020) Prognostic stratification of clinical and molecular epithelioid hemangioendothelioma subsets. Mod. Pathol. 33, 591–602 10.1038/s41379-019-0368-8 31537895 PMC7228463

[113] Bosic M., Kirchner M., Brasanac D., Leichsenring J., Lier A., Volckmar A.-L. et al. (2018) Targeted molecular profiling reveals genetic heterogeneity of poromas and porocarcinomas. Pathology 50, 327–332 10.1016/j.pathol.2017.10.011 29269125

[114] Harms P.W., Hovelson D.H., Cani A.K., Omata K., Haller M.J., Wang M.L. et al. (2016) Porocarcinomas harbor recurrent HRAS-activating mutations and tumor suppressor inactivating mutations. Hum. Pathol. 51, 25–31 10.1016/j.humpath.2015.12.015 27067779

[115] Lamar J., Motilal Nehru V. and Weinberg G. (2018) Epithelioid Hemangioendothelioma as a Model of YAP/TAZ-driven cancer: insights from a rare fusion sarcoma. Cancers (Basel) 10, 229 10.3390/cancers10070229 PMC6070876 29996478

[116] Pajtler K.W., Wei Y., Okonechnikov K., Silva P.B.G., Vouri M., Zhang L. et al. (2019) YAP1 subgroup supratentorial ependymoma requires TEAD and nuclear factor I-mediated transcriptional programmes for tumorigenesis. Nat. Commun. 10, 3914 10.1038/s41467-019-11884-5 31477715 PMC6718408

[117] Fukami M., Wada Y., Okada M., Kato F., Katsumata N., Baba T. et al. (2008) Mastermind-like domain-containing 1 (MAMLD1 or CXorf6) transactivates the Hes3 promoter, augments testosterone production, and contains the SF1 target sequence. J. Biol. Chem. 283, 5525–5532 10.1074/jbc.M703289200 18162467

[118] French C.A. (2018) NUT carcinoma: clinicopathologic features, pathogenesis, and treatment. Pathol. Int. 68, 583–595 10.1111/pin.12727 30362654

[119] Sadowski I., Ma J., Triezenberg S. and Ptashne M. (1988) GAL4-VP16 is an unusually potent transcriptional activator. Nature 335, 563–564 10.1038/335563a0 3047590

[120] Courey A.J., Holtzman D.A., Jackson S.P. and Tjian R. (1989) Synergistic activation by the glutamine-rich domains of human transcription factor Sp1. Cell 59, 827–836 10.1016/0092-8674(89)90606-5 2512012

[121] Nishimura Y., Ryo E., Yamazaki N., Yatabe Y. and Mori T. (2021) Cutaneous primary NUT carcinoma with BRD3-NUTM1 fusion. Am. J. Surg. Pathol. 45, 1582–1584 10.1097/PAS.0000000000001801 34482332

[122] Pei T., Li Y., Wang J., Wang H., Liang Y., Shi H. et al. (2015) YAP is a critical oncogene in human cholangiocarcinoma. Oncotarget 6, 17206–17220 10.18632/oncotarget.4043 26015398 PMC4627302

[123] Hiemer S.E., Zhang L., Kartha V.K., Packer T.S., Almershed M., Noonan V. et al. (2015) A YAP/TAZ-regulated molecular signature is associated with oral squamous cell carcinoma. Mol. Cancer Res. 13, 957–968 10.1158/1541-7786.MCR-14-0580 25794680 PMC4470857

[124] Zhang W., Gao Y., Li F., Tong X., Ren Y., Han X. et al. (2015) YAP promotes malignant progression of Lkb1-deficient lung adenocarcinoma through downstream regulation of Survivin. Cancer Res. 75, 4450–4457 10.1158/0008-5472.CAN-14-3396 26363011

[125] Zanconato F., Forcato M., Battilana G., Azzolin L., Quaranta E., Bodega B. et al. (2015) Genome-wide association between YAP/TAZ/TEAD and AP-1 at enhancers drives oncogenic growth. Nat. Cell Biol. 17, 1218–1227 10.1038/ncb3216 26258633 PMC6186417

[126] Liu X., Li H., Rajurkar M., Li Q., Cotton J.L., Ou J. et al. (2016) Tead and AP1 coordinate transcription and motility. Cell Rep. 14, 1169–1180 10.1016/j.celrep.2015.12.104 26832411 PMC4749442

[127] Hansen C.G., Ng Y.L.D., Lam W.-L.M., Plouffe S.W. and Guan K.-L. (2015) The Hippo pathway effectors YAP and TAZ promote cell growth by modulating amino acid signaling to mTORC1. Cell Res. 25, 1299–1313 10.1038/cr.2015.140 26611634 PMC4670996

[128] Cox A.G., Tsomides A., Yimlamai D., Hwang K.L., Miesfeld J., Galli G.G. et al. (2018) Yap regulates glucose utilization and sustains nucleotide synthesis to enable organ growth. EMBO J. 37, e100294 10.15252/embj.2018100294 30348863 PMC6236334

[129] Watt K.I., Henstridge D.C., Ziemann M., Sim C.B., Montgomery M.K., Samocha-Bonet D. et al. (2021) Yap regulates skeletal muscle fatty acid oxidation and adiposity in metabolic disease. Nat. Commun. 12, 2887 10.1038/s41467-021-23240-7 34001905 PMC8129430

[130] Bergers G. and Fendt S.-M. (2021) The metabolism of cancer cells during metastasis. Nat. Rev. Cancer 21, 162–180 10.1038/s41568-020-00320-2 33462499 PMC8733955

[131] Martínez-Reyes I. and Chandel N.S. (2021) Cancer metabolism: looking forward. Nat. Rev. Cancer 21, 669–680 10.1038/s41568-021-00378-6 34272515

[132] Lunt S.Y. and Vander Heiden M.G. (2011) Aerobic glycolysis: meeting the metabolic requirements of cell proliferation. Annu. Rev. Cell Dev. Biol. 27, 441–464 10.1146/annurev-cellbio-092910-154237 21985671

[133] Yamaguchi H. and Taouk G.M. (2020) A potential role of YAP/TAZ in the interplay between metastasis and metabolic alterations. Front. Oncol. 10, 928 10.3389/fonc.2020.00928 32596154 PMC7300268

[134] Cosset É, Ilmjärv S., Dutoit V., Elliott K., von Schalscha T., Camargo M.F. et al. (2017) Glut3 addiction is a druggable vulnerability for a molecularly defined subpopulation of glioblastoma. Cancer Cell 32, 856.e5–868.e5 10.1016/j.ccell.2017.10.016 29198914 PMC5730343

[135] Warburg O. (1956) On respiratory impairment in cancer cells. Science 124, 269–270 10.1126/science.124.3215.269 13351639

[136] Enzo E., Santinon G., Pocaterra A., Aragona M., Bresolin S., Forcato M. et al. (2015) Aerobic glycolysis tunes YAP/TAZ transcriptional activity. EMBO J. 34, 1349–1370 10.15252/embj.201490379 25796446 PMC4491996

[137] Zhang X., Zhao H., Li Y., Xia D., Yang L., Ma Y. et al. (2018) The role of YAP/TAZ activity in cancer metabolic reprogramming. Mol. Cancer 17, 134 10.1186/s12943-018-0882-1 30176928 PMC6122186

[138] Zheng Y. and Pan D. (2019) The Hippo signaling pathway in development and disease. Dev. Cell 50, 264–282 10.1016/j.devcel.2019.06.003 31386861 PMC6748048

[139] Davis J.R. and Tapon N. (2019) Hippo signalling during development. Development 146, dev167106 10.1242/dev.167106 31527062 PMC7100553

[140] Wu L. and Yang X. (2018) Targeting the Hippo pathway for breast cancer therapy. Cancers (Basel) 10, 422 10.3390/cancers10110422 PMC6266939 30400599

[141] Salem O. and Hansen C.G. (2019) The Hippo pathway in prostate cancer. Cells 8, 370 10.3390/cells8040370 PMC6523349 31018586

[142] Waldmeier L., Meyer-Schaller N., Diepenbruck M. and Christofori G. (2012) Py2T murine breast cancer cells, a versatile model of TGFβ-induced EMT in vitro and in vivo. PLoS ONE 7, e48651 10.1371/journal.pone.0048651 23144919 PMC3492491

[143] Diepenbruck M., Waldmeier L., Ivanek R., Berninger P., Arnold P., van Nimwegen E. et al. (2014) Tead2 expression levels control the subcellular distribution of Yap and Taz, zyxin expression and epithelial-mesenchymal transition. J. Cell Sci. 127, 1523–1536 10.1242/jcs.139865 24554433

[144] Santoro R., Zanotto M., Carbone C., Piro G., Tortora G. and Melisi D. (2018) MEKK3 sustains EMT and stemness in pancreatic cancer by regulating YAP and TAZ transcriptional activity. Anticancer Res. 38, 1937–1946 29599309 10.21873/anticanres.12431

[145] Castellan M., Guarnieri A., Fujimura A., Zanconato F., Battilana G., Panciera T. et al. (2021) Single-cell analyses reveal YAP/TAZ as regulators of stemness and cell plasticity in glioblastoma. Nat. Cancer 2, 174–188 10.1038/s43018-020-00150-z 33644767 PMC7116831

[146] Ellis P., Fagan B.M., Magness S.T., Hutton S., Taranova O., Hayashi S. et al. (2004) SOX2, a persistent marker for multipotential neural stem cells derived from embryonic stem cells, the embryo or the adult. Dev. Neurosci. 26, 148–165 10.1159/000082134 15711057

[147] Ben-Porath I., Thomson M.W., Carey V.J., Ge R., Bell G.W., Regev A. et al. (2008) An embryonic stem cell-like gene expression signature in poorly differentiated aggressive human tumors. Nat. Genet. 40, 499–507 10.1038/ng.127 18443585 PMC2912221

[148] Basu-Roy U., Bayin N.S., Rattanakorn K., Han E., Placantonakis D.G., Mansukhani A. et al. (2015) Sox2 antagonizes the Hippo pathway to maintain stemness in cancer cells. Nat. Commun. 6, 6411 10.1038/ncomms7411 25832504 PMC4429898

[149] Brandt Z.J., Echert A.E., Bostrom J.R., North P.N. and Link B.A. (2020) Core Hippo pathway components act as a brake on Yap/Taz in the development and maintenance of the biliary network. Development 147, 10.1242/dev.184242 PMC7328147 32439761

[150] Sadler K.C., Amsterdam A., Soroka C., Boyer J. and Hopkins N. (2005) A genetic screen in zebrafish identifies the mutants vps18, nf2 and foie gras as models of liver disease. Development 132, 3561–3572 10.1242/dev.01918 16000385

[151] Cox A.G., Hwang K.L., Brown K.K., Evason K.J., Beltz S., Tsomides A. et al. (2016) Yap reprograms glutamine metabolism to increase nucleotide biosynthesis and enable liver growth. Nat. Cell Biol. 18, 886–896 10.1038/ncb3389 27428308 PMC4990146

[152] Dong J., Feldmann G., Huang J., Wu S., Zhang N., Comerford S.A. et al. (2007) Elucidation of a universal size-control mechanism in Drosophila and mammals. Cell 130, 1120–1133 10.1016/j.cell.2007.07.019 17889654 PMC2666353

[153] Yuan W.-C., Pepe-Mooney B., Galli G.G., Dill M.T., Huang H.-T., Hao M. et al. (2018) NUAK2 is a critical YAP target in liver cancer. Nat. Commun. 9, 4834 10.1038/s41467-018-07394-5 30446657 PMC6240092

[154] Pei T., Li Y., Wang J., Wang H., Liang Y., Shi H. et al. (2015) YAP is a critical oncogene in human cholangiocarcinoma. Oncotarget 6, 17206–17220 10.18632/oncotarget.4043 26015398 PMC4627302

[155] Park J., Kim J.S., Nahm J.H., Kim S.-K., Lee D.-H. and Lim D.-S. (2020) WWC1 and NF2 prevent the development of intrahepatic cholangiocarcinoma by regulating YAP/TAZ activity through LATS in mice. Mol. Cells 43, 491–499 32451369 10.14348/molcells.2020.0093 PMC7264477

[156] Hyun J., Al Abo M., Dutta R.K., Oh S.H., Xiang K., Zhou X. et al. (2021) Dysregulation of the ESRP2-NF2-YAP/TAZ axis promotes hepatobiliary carcinogenesis in non-alcoholic fatty liver disease. J. Hepatol. 75, 623–633 10.1016/j.jhep.2021.04.033 33964370 PMC8380690

[157] Zhou Y., Wang Y., Zhou W., Chen T., Wu Q., Chutturghoon V.K. et al. (2019) YAP promotes multi-drug resistance and inhibits autophagy-related cell death in hepatocellular carcinoma via the RAC1-ROS-mTOR pathway. Cancer Cell Int. 19, 179 10.1186/s12935-019-0898-7 31337986 PMC6626386

[158] Spitz F. and Furlong E.E.M. (2012) Transcription factors: from enhancer binding to developmental control. Nat. Rev. Genet. 13, 613–626 10.1038/nrg3207 22868264

[159] Whyte W.A., Orlando D.A., Hnisz D., Abraham B.J., Lin C.Y., Kagey M.H. et al. (2013) Master transcription factors and mediator establish super-enhancers at key cell identity genes. Cell 153, 307–319 10.1016/j.cell.2013.03.035 23582322 PMC3653129

[160] Di Micco R., Fontanals-Cirera B., Low V., Ntziachristos P., Yuen S.K., Lovell C.D. et al. (2014) Control of embryonic stem cell identity by BRD4-dependent transcriptional elongation of super-enhancer-associated pluripotency genes. Cell Rep. 9, 234–247 10.1016/j.celrep.2014.08.055 25263550 PMC4317728

[161] Adam R.C., Yang H., Rockowitz S., Larsen S.B., Nikolova M., Oristian D.S. et al. (2015) Pioneer factors govern super-enhancer dynamics in stem cell plasticity and lineage choice. Nature 521, 366–370 10.1038/nature14289 25799994 PMC4482136

[162] Blinka S., Reimer M.H., Pulakanti K. and Rao S. (2016) Super-enhancers at the Nanog locus differentially regulate neighboring pluripotency-associated genes. Cell Rep. 17, 19–28 10.1016/j.celrep.2016.09.002 27681417 PMC5111363

[163] Hnisz D., Schuijers J., Lin C.Y., Weintraub A.S., Abraham B.J., Lee T.I. et al. (2015) Convergence of developmental and oncogenic signaling pathways at transcriptional super-enhancers. Mol. Cell 58, 362–370 10.1016/j.molcel.2015.02.014 25801169 PMC4402134

[164] Zanconato F., Battilana G., Forcato M., Filippi L., Azzolin L., Manfrin A. et al. (2018) Transcriptional addiction in cancer cells is mediated by YAP/TAZ through BRD4. Nat. Med. 24, 1599–1610 10.1038/s41591-018-0158-8 30224758 PMC6181206

[165] Hnisz D., Shrinivas K., Young R.A., Chakraborty A.K. and Sharp P.A. (2017) A phase separation model for transcriptional control. Cell 169, 13–23 10.1016/j.cell.2017.02.007 28340338 PMC5432200

[166] Hyman A.A., Weber C.A. and Jülicher F. (2014) Liquid-liquid phase separation in biology. Annu. Rev. Cell Dev. Biol. 30, 39–58 10.1146/annurev-cellbio-100913-013325 25288112

[167] Rippe K. (2021) Liquid-liquid phase separation in chromatin. Cold Spring Harb. Perspect. Biol. a040683 10.1101/cshperspect.a040683 34127447 PMC8805649

[168] Cai D., Feliciano D., Dong P., Flores E., Gruebele M., Porat-Shliom N. et al. (2019) Phase separation of YAP reorganizes genome topology for long-term YAP target gene expression. Nat. Cell Biol. 21, 1578–1589 10.1038/s41556-019-0433-z 31792379 PMC8259329

[169] Sun X., Ren Z., Cun Y., Zhao C., Huang X., Zhou J. et al. (2020) Hippo-YAP signaling controls lineage differentiation of mouse embryonic stem cells through modulating the formation of super-enhancers. Nucleic Acids Res. 48, 7182–7196 10.1093/nar/gkaa482 32510157 PMC7367178

[170] Harmar A.J., Hills R.A., Rosser E.M., Jones M., Buneman O.P., Dunbar D.R. et al. (2009) IUPHAR-DB: the IUPHAR database of G protein-coupled receptors and ion channels. Nucleic Acids Res. 37, D680–D685 10.1093/nar/gkn728 18948278 PMC2686540

[171] Bhattacharya M., Babwah A.V. and Ferguson S.S.G. (2004) Small GTP-binding protein-coupled receptors. Biochem. Soc. Trans. 32, 1040–1044 10.1042/BST0321040 15506958

[172] Pierce K.L., Premont R.T. and Lefkowitz R.J. (2002) Seven-transmembrane receptors. Nat. Rev. Mol. Cell Biol. 3, 639–650 10.1038/nrm908 12209124

[173] Gilman A.G. (1987) G proteins: transducers of receptor-generated signals. Annu. Rev. Biochem. 56, 615–649 10.1146/annurev.bi.56.070187.003151 3113327

[174] O’Hayre M., Vázquez-Prado J., Kufareva I., Stawiski E.W., Handel T.M., Seshagiri S. et al. (2013) The emerging mutational landscape of G proteins and G-protein-coupled receptors in cancer. Nat. Rev. Cancer 13, 412–424 10.1038/nrc3521 23640210 PMC4068741

[175] Raimondi F., Inoue A., Kadji F.M.N., Shuai N., Gonzalez J.-C., Singh G. et al. (2019) Rare, functional, somatic variants in gene families linked to cancer genes: GPCR signaling as a paradigm. Oncogene 38, 6491–6506 10.1038/s41388-019-0895-2 31337866 PMC6756116

[176] de Lange M.J., Razzaq L., Versluis M., Verlinde S., Dogrusöz M., Böhringer S. et al. (2015) Distribution of GNAQ and GNA11 mutation signatures in uveal melanoma points to a light dependent mutation mechanism. PLoS ONE 10, e0138002 10.1371/journal.pone.0138002 26368812 PMC4569098

[177] Van Raamsdonk C.D., Bezrookove V., Green G., Bauer J., Gaugler L., O’Brien J.M. et al. (2009) Frequent somatic mutations of GNAQ in uveal melanoma and blue naevi. Nature 457, 599–602 10.1038/nature07586 19078957 PMC2696133

[178] Yu F.-X., Luo J., Mo J.-S., Liu G., Kim Y.C., Meng Z. et al. (2014) Mutant Gq/11 promote uveal melanoma tumorigenesis by activating YAP. Cancer Cell 25, 822–830 10.1016/j.ccr.2014.04.017 24882516 PMC4075337

[179] Chen M., Towers L.N. and O’Connor K.L. (2007) LPA2 (EDG4) mediates Rho-dependent chemotaxis with lower efficacy than LPA1 (EDG2) in breast carcinoma cells. Am. J. Physiol. Cell Physiol. 292, C1927–C1933 10.1152/ajpcell.00400.2006 17496233

[180] Bian D., Mahanivong C., Yu J., Frisch S.M., Pan Z.K., Ye R.D. et al. (2006) The G12/13-RhoA signaling pathway contributes to efficient lysophosphatidic acid-stimulated cell migration. Oncogene 25, 2234–2244 10.1038/sj.onc.1209261 16301993

[181] Regué L., Mou F. and Avruch J. (2013) G protein-coupled receptors engage the mammalian Hippo pathway through F-actin. Bioessays 35, 430–435 10.1002/bies.201200163 23450633 PMC4092039

[182] Gao J., He L., Zhou L., Jing Y., Wang F., Shi Y. et al. (2020) Mechanical force regulation of YAP by F-actin and GPCR revealed by super-resolution imaging. Nanoscale 12, 2703–2714 10.1039/C9NR09452K 31950964

[183] Whitehead I.P., Zohn I.E. and Der C.J. (2001) Rho GTPase-dependent transformation by G protein-coupled receptors. Oncogene 20, 1547–1555 10.1038/sj.onc.1204188 11313901

[184] Horiuchi A., Imai T., Wang C., Ohira S., Feng Y., Nikaido T. et al. (2003) Up-regulation of small GTPases, RhoA and RhoC, is associated with tumor progression in ovarian carcinoma. Lab Investig. 83, 861–870 10.1097/01.LAB.0000073128.16098.31 12808121

[185] Faried A., Faried L.S., Usman N., Kato H. and Kuwano H. (2007) Clinical and prognostic significance of RhoA and RhoC gene expression in esophageal squamous cell carcinoma. Ann. Surg. Oncol. 14, 3593–3601 10.1245/s10434-007-9562-x 17896152

[186] Bellizzi A., Mangia A., Chiriatti A., Petroni S., Quaranta M., Schittulli F. et al. (2008) RhoA protein expression in primary breast cancers and matched lymphocytes is associated with progression of the disease. Int. J. Mol. Med. 22, 25–31 10.3892/ijmm.22.1.25 18575772

[187] Kamai T., Yamanishi T., Shirataki H., Takagi K., Asami H., Ito Y. et al. (2004) Overexpression of RhoA, Rac1, and Cdc42 GTPases is associated with progression in testicular cancer. Clin. Cancer Res. 10, 4799–4805 10.1158/1078-0432.CCR-0436-03 15269155

[188] Xia M. and Land H. (2007) Tumor suppressor p53 restricts Ras stimulation of RhoA and cancer cell motility. Nat. Struct. Mol. Biol. 14, 215–223 10.1038/nsmb1208 17310253

[189] Timpson P., McGhee E.J., Morton J.P., von Kriegsheim A., Schwarz J.P., Karim S.A. et al. (2011) Spatial regulation of RhoA activity during pancreatic cancer cell invasion driven by mutant p53. Cancer Res. 71, 747–757 10.1158/0008-5472.CAN-10-2267 21266354 PMC3033324

[190] Kim D.K., Kim E.K., Jung D.-W. and Kim J. (2019) Cytoskeletal alteration modulates cancer cell invasion through RhoA-YAP signaling in stromal fibroblasts. PLoS ONE 14, e0214553 10.1371/journal.pone.0214553 30921404 PMC6438594

[191] Yu O.M., Benitez J.A., Plouffe S.W., Ryback D., Klein A., Smith J. et al. (2018) YAP and MRTF-A, transcriptional co-activators of RhoA-mediated gene expression, are critical for glioblastoma tumorigenicity. Oncogene 37, 5492–5507 10.1038/s41388-018-0301-5 29887596 PMC6195840

[192] Cai H. and Xu Y. (2013) The role of LPA and YAP signaling in long-term migration of human ovarian cancer cells. Cell Commun. Signal. 11, 31 10.1186/1478-811X-11-31 23618389 PMC3655373

[193] Montaner S., Sodhi A., Molinolo A., Bugge T.H., Sawai E.T., He Y. et al. (2003) Endothelial infection with KSHV genes in vivo reveals that vGPCR initiates Kaposi’s sarcomagenesis and can promote the tumorigenic potential of viral latent genes. Cancer Cell. 3, 23–36 10.1016/S1535-6108(02)00237-4 12559173

[194] Liu G., Yu F.-X., Kim Y.C., Meng Z., Naipauer J., Looney D.J. et al. (2015) Kaposi sarcoma-associated herpesvirus promotes tumorigenesis by modulating the Hippo pathway. Oncogene 34, 3536–3546 10.1038/onc.2014.281 25195862 PMC4721508

[195] Prior I.A., Lewis P.D. and Mattos C. (2012) A comprehensive survey of Ras mutations in cancer. Cancer Res. 72, 2457–2467 10.1158/0008-5472.CAN-11-2612 22589270 PMC3354961

[196] Prior I.A., Hood F.E. and Hartley J.L. (2020) The frequency of Ras mutations in cancer. Cancer Res. 80, 2969–2974 10.1158/0008-5472.CAN-19-3682 32209560 PMC7367715

[197] Pascual J., Jacobs J., Sansores-Garcia L., Natarajan M., Zeitlinger J., Aerts S. et al. (2017) Hippo reprograms the transcriptional response to Ras signaling. Dev. Cell 42, 667.e4–680.e4 10.1016/j.devcel.2017.08.013 28950103

[198] Shao D.D., Xue W., Krall E.B., Bhutkar A., Piccioni F., Wang X. et al. (2014) KRAS and YAP1 converge to regulate EMT and tumor survival. Cell 158, 171–184 10.1016/j.cell.2014.06.004 24954536 PMC4110062

[199] Muzumdar M.D., Chen P.-Y., Dorans K.J., Chung K.M., Bhutkar A., Hong E. et al. (2017) Survival of pancreatic cancer cells lacking KRAS function. Nat. Commun. 8, 1090 10.1038/s41467-017-00942-5 29061961 PMC5653666

[200] Eser S., Schnieke A., Schneider G. and Saur D. (2014) Oncogenic KRAS signalling in pancreatic cancer. Br. J. Cancer 111, 817–822 10.1038/bjc.2014.215 24755884 PMC4150259

[201] Tu B., Yao J., Ferri-Borgogno S., Zhao J., Chen S., Wang Q. et al. (2019) YAP1 oncogene is a context-specific driver for pancreatic ductal adenocarcinoma. JCI Insight 4, e130811 10.1172/jci.insight.130811 PMC6948828 31557131

[202] Salcedo Allende M.T., Zeron-Medina J., Hernandez J., Macarulla T., Balsells J., Merino X. et al. (2017) Overexpression of Yes associated protein 1, an independent prognostic marker in patients with pancreatic ductal adenocarcinoma, correlated with liver metastasis and poor prognosis. Pancreas 46, 913–920 10.1097/MPA.0000000000000867 28697132

[203] Cottini F., Hideshima T., Xu C., Sattler M., Dori M., Agnelli L. et al. (2014) Rescue of Hippo coactivator YAP1 triggers DNA damage-induced apoptosis in hematological cancers. Nat. Med. 20, 599–606 10.1038/nm.3562 24813251 PMC4057660

[204] Strano S., Monti O., Pediconi N., Baccarini A., Fontemaggi G., Lapi E. et al. (2005) The transcriptional coactivator Yes-associated protein drives p73 gene-target specificity in response to DNA damage. Mol. Cell 18, 447–459 10.1016/j.molcel.2005.04.008 15893728

[205] Rudin C.M., Poirier J.T., Byers L.A., Dive C., Dowlati A., George J. et al. (2019) Molecular subtypes of small cell lung cancer: a synthesis of human and mouse model data. Nat. Rev. Cancer 19, 289–297 10.1038/s41568-019-0133-9 30926931 PMC6538259

[206] Carter S.L., Negrini M., Baffa R., Gillum D.R., Rosenberg A.L., Schwartz G.F. et al. (1994) Loss of heterozygosity at 11q22-q23 in breast cancer. Cancer Res. 54, 6270–6274 7954477

[207] Yuan M., Tomlinson V., Lara R., Holliday D., Chelala C., Harada T. et al. (2008) Yes-associated protein (YAP) functions as a tumor suppressor in breast. Cell Death Differ. 15, 1752–1759 10.1038/cdd.2008.108 18617895

[208] Fan H., Wang X., Li W., Shen M., Wei Y., Zheng H. et al. (2020) ASB13 inhibits breast cancer metastasis through promoting SNAI2 degradation and relieving its transcriptional repression of YAP. Genes Dev. 34, 1359–1372 10.1101/gad.339796.120 32943576 PMC7528707

[209] Zhu M., Peng R., Liang X., Lan Z., Tang M., Hou P. et al. (2021) P4HA2-induced prolyl hydroxylation suppresses YAP1-mediated prostate cancer cell migration, invasion, and metastasis. Oncogene 40, 6049–6056 10.1038/s41388-021-02000-3 34471235 PMC8526415

[210] Cheng S., Prieto-Dominguez N., Yang S., Connelly Z.M., StPierre S., Rushing B. et al. (2020) The expression of YAP1 is increased in high-grade prostatic adenocarcinoma but is reduced in neuroendocrine prostate cancer. Prostate Cancer Prostatic Dis. 23, 661–669 10.1038/s41391-020-0229-z 32313141 PMC7572469

[211] Finch-Edmondson M.L., Strauss R.P., Passman A.M., Sudol M., Yeoh G.C. and Callus B.A. (2015) TAZ protein accumulation is negatively regulated by YAP abundance in mammalian cells. J. Biol. Chem. 290, 27928–27938 10.1074/jbc.M115.692285 26432639 PMC4646034

[212] Moroishi T., Park H.W., Qin B., Chen Q., Meng Z., Plouffe S.W. et al. (2015) A YAP/TAZ-induced feedback mechanism regulates Hippo pathway homeostasis. Genes Dev. 29, 1271–1284 10.1101/gad.262816.115 26109050 PMC4495398

[213] Thériault B.L., Dimaras H., Gallie B.L. and Corson T.W. (2014) The genomic landscape of retinoblastoma: a review. Clin. Experiment. Ophthalmol. 42, 33–52 10.1111/ceo.12132 24433356 PMC3896868

[214] Pearson J.D., Huang K., Pacal M., McCurdy S.R., Lu S., Aubry A. et al. (2021) Binary pan-cancer classes with distinct vulnerabilities defined by pro- or anti-cancer YAP/TEAD activity. Cancer Cell 39, 1115–1134, e12. 10.1016/j.ccell.2021.06.016 34270926 PMC8981970

[215] Moya I.M., Castaldo S.A., Van den Mooter L., Soheily S., Sansores-Garcia L., Jacobs J. et al. (2019) Peritumoral activation of the Hippo pathway effectors YAP and TAZ suppresses liver cancer in mice. Science (80-) 366, 1029–1034 10.1126/science.aaw9886 31754005

[216] Messmer K.J. and Abel S.R. (2001) Verteporfin for age-related macular degeneration. Ann. Pharmacother. 35, 1593–1598 10.1345/aph.10365 11793628

[217] Liu-Chittenden Y., Huang B., Shim J.S., Chen Q., Lee S.-J., Anders R.A. et al. (2012) Genetic and pharmacological disruption of the TEAD-YAP complex suppresses the oncogenic activity of YAP. Genes Dev. 26, 1300–1305 10.1101/gad.192856.112 22677547 PMC3387657

[218] Zhang H., Ramakrishnan S.K., Triner D., Centofanti B., Maitra D., Győrffy B. et al. (2015) Tumor-selective proteotoxicity of verteporfin inhibits colon cancer progression independently of YAP1. Sci. Signal. 8, ra98 10.1126/scisignal.aac5418 26443705 PMC4818013

[219] Dasari V.R., Mazack V., Feng W., Nash J., Carey D.J. and Gogoi R. (2017) Verteporfin exhibits YAP-independent anti-proliferative and cytotoxic effects in endometrial cancer cells. Oncotarget 8, 28628–28640 10.18632/oncotarget.15614 28404908 PMC5438678

[220] Smith S.A., Sessions R.B., Shoemark D.K., Williams C., Ebrahimighaei R., McNeill M.C. et al. (2019) Antiproliferative and antimigratory effects of a novel YAP-TEAD interaction inhibitor identified using in silico molecular docking. J. Med. Chem. 62, 1291–1305 10.1021/acs.jmedchem.8b01402 30640473 PMC6701825

[221] Bum-Erdene K., Zhou D., Gonzalez-Gutierrez G., Ghozayel M.K., Si Y., Xu D. et al. (2019) Small-molecule covalent modification of conserved cysteine leads to allosteric inhibition of the TEAD⋅Yap protein-protein interaction. Cell Chem. Biol. 26, 378–389, e13. 10.1016/j.chembiol.2018.11.010 30581134

[222] Kunig V.B.K., Potowski M., Akbarzadeh M., Klika Škopić M., Santos Smith D., Arendt L. et al. (2020) TEAD-YAP interaction inhibitors and MDM2 binders from DNA‐encoded indole‐focused Ugi peptidomimetics. Angew Chemie 132, 20518–20522 10.1002/ange.202006280 PMC7689693 32537835

[223] Holden J.K., Crawford J.J., Noland C.L., Schmidt S., Zbieg J.R., Lacap J.A. et al. (2020) Small molecule dysregulation of TEAD lipidation induces a dominant-negative inhibition of Hippo pathway signaling. Cell Rep. 31, 107809 10.1016/j.celrep.2020.107809 32579935

[224] Tang T.T., Konradi A.W., Feng Y., Peng X., Ma M., Li J. et al. (2021) Small molecule inhibitors of TEAD auto-palmitoylation selectively inhibit proliferation and tumor growth of NF2-deficient mesothelioma. Mol. Cancer Ther. 20, 986–998 10.1158/1535-7163.MCT-20-0717 33850002

[225] Lu T., Li Y., Lu W., Spitters T., Fang X., Wang J. et al. (2021) Discovery of a subtype-selective, covalent inhibitor against palmitoylation pocket of TEAD3. Acta Pharm. Sin. B. 11, 3206–3219 10.1016/j.apsb.2021.04.015 34729310 PMC8546857

[226] Knight J.F., Shepherd C.J., Rizzo S., Brewer D., Jhavar S., Dodson A.R. et al. (2008) TEAD1 and c-Cbl are novel prostate basal cell markers that correlate with poor clinical outcome in prostate cancer. Br. J. Cancer 99, 1849–1858 10.1038/sj.bjc.6604774 19002168 PMC2600693

[227] Zhang W., Li J., Wu Y., Ge H., Song Y., Wang D. et al. (2018) TEAD4 overexpression promotes epithelial-mesenchymal transition and associates with aggressiveness and adverse prognosis in head neck squamous cell carcinoma. Cancer Cell Int. 18, 178 10.1186/s12935-018-0675-z 30459528 PMC6233371

[228] Joo J., Cho S., Rou W., Kim J., Kang S., Lee E. et al. (2020) TEAD2 as a novel prognostic factor for hepatocellular carcinoma. Oncol. Rep. 43, 1785–1796 10.3892/or.2020.7578 32323824 PMC7160555

[229] Fan F., He Z., Kong L.-L., Chen Q., Yuan Q., Zhang S. et al. (2016) Pharmacological targeting of kinases MST1 and MST2 augments tissue repair and regeneration. Sci. Transl. Med. 8, 352ra108 10.1126/scitranslmed.aaf2304 27535619

[230] Xu C.M., Liu W.W., Liu C.J., Wen C., Lu H.F. and Wan F.S. (2013) Mst1 overexpression inhibited the growth of human non-small cell lung cancer in vitro and in vivo. Cancer Gene Ther. 20, 453–460 10.1038/cgt.2013.40 23928732

[231] Cui J., Zhou Z., Yang H., Jiao F., Li N., Gao Y. et al. (2019) MST1 suppresses pancreatic cancer progression via ROS-induced pyroptosis. Mol. Cancer Res. 17, 1316–1325 10.1158/1541-7786.MCR-18-0910 30796177

[232] Singh K., Pruski M.A., Polireddy K., Jones N.C., Chen Q., Yao J. et al. (2020) Mst1/2 kinases restrain transformation in a novel transgenic model of Ras driven non-small cell lung cancer. Oncogene 39, 1152–1164 10.1038/s41388-019-1031-z 31570790

[233] Jumper J., Evans R., Pritzel A., Green T., Figurnov M., Ronneberger O. et al. (2021) Highly accurate protein structure prediction with AlphaFold. Nature 596, 583–589 10.1038/s41586-021-03819-2 34265844 PMC8371605

[234] Bhullar K.S., Lagarón N.O., McGowan E.M., Parmar I., Jha A., Hubbard B.P. et al. (2018) Kinase-targeted cancer therapies: progress, challenges and future directions. Mol. Cancer 17, 48 10.1186/s12943-018-0804-2 29455673 PMC5817855

[235] Cohen P., Cross D. and Jänne P.A. (2021) Kinase drug discovery 20 years after imatinib: progress and future directions. Nat. Rev. Drug Discov. 20, 551–569 10.1038/s41573-021-00195-4 34002056 PMC8127496

[236] Polekhina G., Gupta A., Michell B.J., van Denderen B., Murthy S., Feil S.C. et al. (2003) AMPK β subunit targets metabolic stress sensing to glycogen. Curr. Biol. 13, 867–871 10.1016/S0960-9822(03)00292-6 12747837

[237] DeFronzo R.A. and Goodman A.M. (1995) Efficacy of metformin in patients with non-insulin-dependent diabetes mellitus. N. Engl. J. Med. 333, 541–549 10.1056/NEJM199508313330902 7623902

[238] Steinberg G.R. and Carling D. (2019) AMP-activated protein kinase: the current landscape for drug development. Nat. Rev. Drug Discov. 18, 527–551 10.1038/s41573-019-0019-2 30867601

[239] Zadra G., Photopoulos C., Tyekucheva S., Heidari P., Weng Q.P., Fedele G. et al. (2014) A novel direct activator of AMPK inhibits prostate cancer growth by blocking lipogenesis. EMBO Mol. Med. 6, 519–538 10.1002/emmm.201302734 24497570 PMC3992078

[240] O’Brien A.J., Villani L.A., Broadfield L.A., Houde V.P., Galic S., Blandino G. et al. (2015) Salicylate activates AMPK and synergizes with metformin to reduce the survival of prostate and lung cancer cells ex vivo through inhibition of de novo lipogenesis. Biochem. J. 469, 177–187 10.1042/BJ20150122 25940306

[241] Griss T., Vincent E.E., Egnatchik R., Chen J., Ma E.H., Faubert B. et al. (2015) Metformin antagonizes cancer cell proliferation by suppressing mitochondrial-dependent biosynthesis. PLoS Biol. 13, e1002309 10.1371/journal.pbio.1002309 26625127 PMC4666657

[242] Sriram K. and Insel P.A. (2018) G protein-coupled receptors as targets for approved drugs: how many targets and how many drugs? Mol. Pharmacol. 93, 251–258 10.1124/mol.117.111062 29298813 PMC5820538

[243] Cornwell A.C. and Feigin M.E. (2020) Unintended effects of GPCR-targeted drugs on the cancer phenotype. Trends Pharmacol. Sci. 41, 1006–1022 10.1016/j.tips.2020.10.001 33198923 PMC7672258

[244] Nieto Gutierrez A. and McDonald P.H. (2018) GPCRs: emerging anti-cancer drug targets. Cell. Signal. 41, 65–74 10.1016/j.cellsig.2017.09.005 28931490

[245] Feng X., Arang N., Rigiracciolo D.C., Lee J.S., Yeerna H., Wang Z. et al. (2019) A platform of synthetic lethal gene interaction networks reveals that the GNAQ uveal melanoma oncogene controls the Hippo pathway through FAK. Cancer Cell. 35, 457.e5–472.e5 10.1016/j.ccell.2019.01.009 30773340 PMC6737937

[246] Murphy K.J., Reed D.A., Vennin C., Conway J.R.W., Nobis M., Yin J.X. et al. (2021) Intravital imaging technology guides FAK-mediated priming in pancreatic cancer precision medicine according to Merlin status. Sci. Adv. 7, eabh0363 10.1126/sciadv.abh0363 34586840 PMC8480933

[247] Filippakopoulos P., Qi J., Picaud S., Shen Y., Smith W.B., Fedorov O. et al. (2010) Selective inhibition of BET bromodomains. Nature 468, 1067–1073 10.1038/nature09504 20871596 PMC3010259

[248] Delmore J.E., Issa G.C., Lemieux M.E., Rahl P.B., Shi J., Jacobs H.M. et al. (2011) BET bromodomain inhibition as a therapeutic strategy to target c-Myc. Cell 146, 904–917 10.1016/j.cell.2011.08.017 21889194 PMC3187920

[249] Leal A.S., Williams C.R., Royce D.B., Pioli P.A., Sporn M.B. and Liby K.T. (2017) Bromodomain inhibitors, JQ1 and I-BET 762, as potential therapies for pancreatic cancer. Cancer Lett. 394, 76–87 10.1016/j.canlet.2017.02.021 28254412

[250] Hernandez-Quiles M., Broekema M.F. and Kalkhoven E. (2021) PPARgamma in metabolism, immunity, and cancer: unified and diverse mechanisms of action. Front. Endocrinol. (Lausanne) 12, 624112 10.3389/fendo.2021.624112 33716977 PMC7953066

[251] Basu-Roy U., Han E., Rattanakorn K., Gadi A., Verma N., Maurizi G. et al. (2016) PPARγ agonists promote differentiation of cancer stem cells by restraining YAP transcriptional activity. Oncotarget 7, 60954–60970 10.18632/oncotarget.11273 27528232 PMC5308629

[252] Della Chiara G., Gervasoni F., Fakiola M., Godano C., D’Oria C., Azzolin L. et al. (2021) Epigenomic landscape of human colorectal cancer unveils an aberrant core of pan-cancer enhancers orchestrated by YAP/TAZ. Nat. Commun. 12, 2340 10.1038/s41467-021-22544-y 33879786 PMC8058065

[253] Guo X., Zhao Y., Yan H., Yang Y., Shen S., Dai X. et al. (2017) Single tumor-initiating cells evade immune clearance by recruiting type II macrophages. Genes Dev. 31, 247–259 10.1101/gad.294348.116 28223311 PMC5358722

[254] Wang G., Lu X., Dey P., Deng P., Wu C.C., Jiang S. et al. (2016) Targeting YAP-dependent MDSC infiltration impairs tumor progression. Cancer Discov. 6, 80–95 10.1158/2159-8290.CD-15-0224 26701088 PMC4707102

[255] Murakami S., Shahbazian D., Surana R., Zhang W., Chen H., Graham G.T. et al. (2017) Yes-associated protein mediates immune reprogramming in pancreatic ductal adenocarcinoma. Oncogene 36, 1232–1244 10.1038/onc.2016.288 27546622 PMC5322249

[256] Lee B.S., Park D.Il., Lee D.H., Lee J.E., Yeo M.-K., Park Y.H. et al. (2017) Hippo effector YAP directly regulates the expression of PD-L1 transcripts in EGFR-TKI-resistant lung adenocarcinoma. Biochem. Biophys. Res. Commun. 491, 493–499 10.1016/j.bbrc.2017.07.007 28684311

[257] Janse van Rensburg H.J., Azad T., Ling M., Hao Y., Snetsinger B., Khanal P. et al. (2018) The Hippo pathway component TAZ promotes immune evasion in human cancer through PD-L1. Cancer Res. 78, 1457–1470 10.1158/0008-5472.CAN-17-3139 29339539

[258] Ishida Y., Agata Y., Shibahara K. and Honjo T. (1992) Induced expression of PD-1, a novel member of the immunoglobulin gene superfamily, upon programmed cell death. EMBO J. 11, 3887–3895 10.1002/j.1460-2075.1992.tb05481.x 1396582 PMC556898

[259] Keir M.E., Liang S.C., Guleria I., Latchman Y.E., Qipo A., Albacker L.A. et al. (2006) Tissue expression of PD-L1 mediates peripheral T cell tolerance. J. Exp. Med. 203, 883–895 10.1084/jem.20051776 16606670 PMC2118286

[260] Houot R., Schultz L.M., Marabelle A. and Kohrt H. (2015) T-cell-based immunotherapy: adoptive cell transfer and checkpoint inhibition. Cancer Immunol. Res. 3, 1115–1122 10.1158/2326-6066.CIR-15-0190 26438444

[261] Ni X., Tao J., Barbi J., Chen Q., Park B.V., Li Z. et al. (2018) YAP is essential for Treg-mediated suppression of antitumor immunity. Cancer Discov. 8, 1026–1043 10.1158/2159-8290.CD-17-1124 29907586 PMC6481611

[262] Farkona S., Diamandis E.P. and Blasutig I.M. (2016) Cancer immunotherapy: the beginning of the end of cancer? BMC Med. 14, 73 10.1186/s12916-016-0623-5 27151159 PMC4858828

[263] Mellman I., Coukos G. and Dranoff G. (2011) Cancer immunotherapy comes of age. Nature 480, 480–489 10.1038/nature10673 22193102 PMC3967235

[264] Barrueto L., Caminero F., Cash L., Makris C., Lamichhane P. and Deshmukh R.R. (2020) Resistance to checkpoint inhibition in cancer immunotherapy. Transl. Oncol. 13, 100738 10.1016/j.tranon.2019.12.010 32114384 PMC7047187

[265] Nguyen C.D.K. and Yi C. (2019) YAP/TAZ signaling and resistance to cancer therapy. Trends Cancer 5, 283–296 10.1016/j.trecan.2019.02.010 31174841 PMC6557283

[266] Liu J., Gao M., Nipper M., Deng J., Sharkey F.E., Johnson R.L. et al. (2019) Activation of the intrinsic fibroinflammatory program in adult pancreatic acinar cells triggered by Hippo signaling disruption. PLoS Biol. 17, e3000418 10.1371/journal.pbio.3000418 31513574 PMC6742234

[267] Zhao B., Ye X., Yu J., Li L., Li W., Li S. et al. (2008) TEAD mediates YAP-dependent gene induction and growth control. Genes Dev. 22, 1962–1971 10.1101/gad.1664408 18579750 PMC2492741

[268] Wang K.-C., Yeh Y.-T., Nguyen P., Limqueco E., Lopez J., Thorossian S. et al. (2016) Flow-dependent YAP/TAZ activities regulate endothelial phenotypes and atherosclerosis. Proc. Natl. Acad. Sci. U.S.A. 113, 11525–11530 10.1073/pnas.1613121113 27671657 PMC5068257

[269] Wang X., Zheng Z., Caviglia J.M., Corey K.E., Herfel T.M., Cai B. et al. (2016) Hepatocyte TAZ/WWTR1 promotes inflammation and fibrosis in nonalcoholic steatohepatitis. Cell Metab. 24, 848–862 10.1016/j.cmet.2016.09.016 28068223 PMC5226184

[270] Taniguchi K., Wu L.-W., Grivennikov S.I., de Jong P.R., Lian I., Yu F.-X. et al. (2015) A gp130-Src-YAP module links inflammation to epithelial regeneration. Nature 519, 57–62 10.1038/nature14228 25731159 PMC4447318

[271] Moroishi T., Hayashi T., Pan W.-W., Fujita Y., Holt M.V., Qin J. et al. (2016) The Hippo pathway kinases LATS1/2 suppress cancer immunity. Cell 167, 1525–1539, e17. 10.1016/j.cell.2016.11.005 27912060 PMC5512418

[272] Panagopoulou M.S., Wark A.W., Birch D.J.S. and Gregory C.D. (2020) Phenotypic analysis of extracellular vesicles: a review on the applications of fluorescence. J. Extracell. Vesicles 9, 1710020 10.1080/20013078.2019.1710020 32002172 PMC6968689

[273] Han L., Lam E.W.-F. and Sun Y. (2019) Extracellular vesicles in the tumor microenvironment: old stories, but new tales. Mol. Cancer 18, 59 10.1186/s12943-019-0980-8 30925927 PMC6441234

[274] Yamauchi T. and Moroishi T. (2019) Hippo pathway in mammalian adaptive immune system. Cells 8, 398 10.3390/cells8050398 PMC6563119 31052239

[275] Holden J. and Cunningham C. (2018) Targeting the Hippo pathway and cancer through the TEAD family of transcription factors. Cancers (Basel) 10, 81 10.3390/cancers10030081 PMC5876656 29558384

[276] Juan W. and Hong W. (2016) Targeting the Hippo signaling pathway for tissue regeneration and cancer therapy. Genes (Basel) 7, 55 10.3390/genes7090055 PMC5042386 27589805

[277] Gobbi G., Donati B., Do Valle I.F., Reggiani F., Torricelli F., Remondini D. et al. (2019) The Hippo pathway modulates resistance to BET proteins inhibitors in lung cancer cells. Oncogene 38, 6801–6817 10.1038/s41388-019-0924-1 31406246

[278] Song S., Li Y., Xu Y., Ma L., Pool Pizzi M., Jin J. et al. (2020) Targeting Hippo coactivator YAP1 through BET bromodomain inhibition in esophageal adenocarcinoma. Mol. Oncol. 14, 1410–1426 10.1002/1878-0261.12667 32175692 PMC7266288

[279] Luo J. and Yu F.-X. (2019) GPCR-Hippo signaling in cancer. Cells 8, 426 10.3390/cells8050426 PMC6563442 31072060

[280] DeRan M., Yang J., Shen C.-H., Peters E.C., Fitamant J., Chan P. et al. (2014) Energy stress regulates Hippo-YAP signaling involving ampk-mediated regulation of angiomotin-like 1 protein. Cell Rep. 9, 495–503 10.1016/j.celrep.2014.09.036 25373897 PMC4223634

[281] Mo J.-S., Meng Z, Kim Y.C., Park H.W., Hansen C.G., Kim S. et al. (2015) Cellular energy stress induces AMPK-mediated regulation of YAP and the Hippo pathway. Nat. Cell Biol. 17, 500–510 10.1038/ncb3111 25751140 PMC4380774

[282] Stancu C. and Sima A. (2001) Statins: mechanism of action and effects. J. Cell. Mol. Med. 5, 378–387 10.1111/j.1582-4934.2001.tb00172.x 12067471 PMC6740083

[283] Wang Z., Wu Y., Wang H., Zhang Y., Mei L., Fang X. et al. (2014) Interplay of mevalonate and Hippo pathways regulates RHAMM transcription via YAP to modulate breast cancer cell motility. Proc. Natl. Acad. Sci. U.S.A. 111, E89–E98 10.1073/pnas.1319190110 24367099 PMC3890879

[284] Sorrentino G., Ruggeri N., Specchia V., Cordenonsi M., Mano M., Dupont S. et al. (2014) Metabolic control of YAP and TAZ by the mevalonate pathway. Nat. Cell Biol. 16, 357–366 10.1038/ncb2936 24658687

[285] Kang J., Jeong S.-M., Shin D.W., Cho M., Cho J.H. and Kim J. (2021) The associations of aspirin, statins, and metformin with lung cancer risk and related mortality: a time-dependent analysis of population-based nationally representative data. J. Thorac. Oncol. 16, 76–88 10.1016/j.jtho.2020.08.021 32950701

[286] Longo J., van Leeuwen J.E., Elbaz M., Branchard E. and Penn L.Z. (2020) Statins as anticancer agents in the era of precision medicine. Clin. Cancer Res. 26, 5791–5800 10.1158/1078-0432.CCR-20-1967 32887721

[287] Shojaee S. and Nana-Sinkam P. (2021) One metformin a day, keeps lung cancer away! Or does it? J. Thorac. Oncol. 16, 11–13 10.1016/j.jtho.2020.10.005 33384055

[288] Sekido Y. (2011) Inactivation of Merlin in malignant mesothelioma cells and the Hippo signaling cascade dysregulation. Pathol. Int. 61, 331–344 10.1111/j.1440-1827.2011.02666.x 21615608

[289] Bethune G., Bethune D., Ridgway N. and Xu Z. (2010) Epidermal growth factor receptor (EGFR) in lung cancer: an overview and update. J. Thorac. Dis. 2, 48–51 22263017 PMC3256436

[290] Sigismund S., Avanzato D. and Lanzetti L. (2018) Emerging functions of the EGFR in cancer. Mol. Oncol. 12, 3–20 10.1002/1878-0261.12155 29124875 PMC5748484

[291] Ando T., Arang N., Wang Z., Costea D.E., Feng X., Goto Y. et al. (2021) EGFR Regulates the Hippo pathway by promoting the tyrosine phosphorylation of MOB1. Commun. Biol. 4, 1237 10.1038/s42003-021-02744-4 34725466 PMC8560880

[292] Hall C.A., Wang R., Miao J., Oliva E., Shen X., Wheeler T. et al. (2010) Hippo pathway effector Yap is an ovarian cancer oncogene. Cancer Res. 70, 8517–8525 10.1158/0008-5472.CAN-10-1242 20947521 PMC2970655

[293] Zhao Y., Khanal P., Savage P., She Y.-M., Cyr T.D. and Yang X. (2014) YAP-induced resistance of cancer cells to antitubulin drugs is modulated by a Hippo-independent pathway. Cancer Res. 74, 4493–4503 10.1158/0008-5472.CAN-13-2712 24812269

[294] Yan L., Cai Q. and Xu Y. (2014) Hypoxic conditions differentially regulate TAZ and YAP in cancer cells. Arch. Biochem. Biophys. 562, 31–36 10.1016/j.abb.2014.07.024 25078107 PMC4197065

[295] Yang S., Zhang L., Liu M., Chong R., Ding S.-J., Chen Y. et al. (2013) CDK1 phosphorylation of YAP promotes mitotic defects and cell motility and is essential for neoplastic transformation. Cancer Res. 73, 6722–6733 10.1158/0008-5472.CAN-13-2049 24101154 PMC3861241

[296] Plouffe S.W., Lin K.C., Moore J.L., Tan F.E., Ma S., Ye Z. et al. (2018) The Hippo pathway effector proteins YAP and TAZ have both distinct and overlapping functions in the cell. J. Biol. Chem. 293, 11230–11240 10.1074/jbc.RA118.002715 29802201 PMC6052207

[297] Cordenonsi M., Zanconato F., Azzolin L., Forcato M., Rosato A., Frasson C. et al. (2011) The Hippo transducer TAZ confers cancer stem cell-related traits on breast cancer cells. Cell 147, 759–772 10.1016/j.cell.2011.09.048 22078877

[298] Bhat K.P.L., Salazar K.L., Balasubramaniyan V., Wani K., Heathcock L., Hollingsworth F. et al. (2011) The transcriptional coactivator TAZ regulates mesenchymal differentiation in malignant glioma. Genes Dev. 25, 2594–2609 10.1101/gad.176800.111 22190458 PMC3248681

[299] Li Z., Wang Y., Zhu Y., Yuan C., Wang D., Zhang W. et al. (2015) The Hippo transducer TAZ promotes epithelial to mesenchymal transition and cancer stem cell maintenance in oral cancer. Mol. Oncol. 9, 1091–1105 10.1016/j.molonc.2015.01.007 25704916 PMC5528756

[300] Li J., Li Z., Wu Y., Wang Y., Wang D., Zhang W. et al. (2019) The Hippo effector TAZ promotes cancer stemness by transcriptional activation of SOX2 in head neck squamous cell carcinoma. Cell Death Dis. 10, 603 10.1038/s41419-019-1838-0 31399556 PMC6689034

[301] Hayashi H., Higashi T., Yokoyama N., Kaida T., Sakamoto K., Fukushima Y. et al. (2015) An imbalance in TAZ and YAP expression in hepatocellular carcinoma confers cancer stem cell-like behaviors contributing to disease progression. Cancer Res. 75, 4985–4997 10.1158/0008-5472.CAN-15-0291 26420216

[302] Miesfeld J.B., Gestri G., Clark B.S., Flinn M.A., Poole R.J., Bader J.R. et al. (2015) Yap and Taz regulate retinal pigment epithelial cell fate. Development 142, 3021–3032 26209646 10.1242/dev.119008 PMC4582179

[303] Yang H., Hall S.R.R., Sun B., Zhao L., Gao Y., Schmid R.A. et al. (2021) NF2 and canonical Hippo-YAP pathway define distinct tumor subsets characterized by different immune deficiency and treatment implications in human pleural mesothelioma. Cancers (Basel) 13, 1561 10.3390/cancers13071561 33805359 PMC8036327

[304] Britschgi A., Duss S., Kim S., Couto J.P., Brinkhaus H., Koren S. et al. (2017) The Hippo kinases LATS1 and 2 control human breast cell fate via crosstalk with ERα. Nature 541, 541–545 10.1038/nature20829 28068668 PMC6726477

[305] Díaz-Martín J., López-García M.Á., Romero-Pérez L., Atienza-Amores M.R., Pecero M.L., Castilla M.Á. et al. (2015) Nuclear TAZ expression associates with the triple-negative phenotype in breast cancer. Endocr. Relat. Cancer 22, 443–454 10.1530/ERC-14-0456 25870251

[306] Kim T., Yang S.-J., Hwang D., Song J., Kim M., Kyum Kim S. et al. (2015) A basal-like breast cancer-specific role for SRF-IL6 in YAP-induced cancer stemness. Nat. Commun. 6, 10186 10.1038/ncomms10186 26671411 PMC4703869

[307] Guo J., Wu Y., Yang L., Du J., Gong K., Chen W. et al. (2017) Repression of YAP by NCTD disrupts NSCLC progression. Oncotarget 8, 2307–2319 10.18632/oncotarget.13668 27903989 PMC5356801

[308] Kim J.M., Kang D.W., Long L.Z., Huang S.-M., Yeo M.-K., Yi E.S. et al. (2011) Differential expression of Yes-associated protein is correlated with expression of cell cycle markers and pathologic TNM staging in non-small-cell lung carcinoma☆. Hum. Pathol. 42, 315–323 10.1016/j.humpath.2010.08.003 21190720

[309] Noguchi S., Saito A., Horie M., Mikami Y., Suzuki H.I., Morishita Y. et al. (2014) An integrative analysis of the tumorigenic role of TAZ in human non-small cell lung cancer. Clin. Cancer Res. 20, 4660–4672 10.1158/1078-0432.CCR-13-3328 24951773

[310] Noto A., De Vitis C., Pisanu M.E., Roscilli G., Ricci G., Catizone A. et al. (2017) Stearoyl-CoA-desaturase 1 regulates lung cancer stemness via stabilization and nuclear localization of YAP/TAZ. Oncogene 36, 4573–4584 10.1038/onc.2017.75 28368399

[311] Zhang Y.-H., Li B., Shen L., Shen Y. and Chen X.-D. (2013) The role and clinical significance of YES-associated protein 1 in human osteosarcoma. Int. J. Immunopathol. Pharmacol. 26, 157–167 10.1177/039463201302600115 23527718

[312] Bouvier C., Macagno N., Nguyen Q., Loundou A., Jiguet-Jiglaire C., Gentet J.-C. et al. (2016) Prognostic value of the Hippo pathway transcriptional coactivators YAP/TAZ and β1-integrin in conventional osteosarcoma. Oncotarget 7, 64702–64710 10.18632/oncotarget.11876 27608849 PMC5323109

[313] Bailey P., Chang D.K., Nones K., Johns A.L., Patch A.-M., Gingras M.-C. et al. (2016) Genomic analyses identify molecular subtypes of pancreatic cancer. Nature 531, 47–52 10.1038/nature16965 26909576

[314] Kuser-Abali G., Alptekin A., Lewis M., Garraway I.P. and Cinar B. (2015) YAP1 and AR interactions contribute to the switch from androgen-dependent to castration-resistant growth in prostate cancer. Nat. Commun. 6, 8126 10.1038/ncomms9126 28230103 PMC5327734

[315] Zhang L., Yang S., Chen X., Stauffer S., Yu F., Lele S.M. et al. (2015) The Hippo pathway effector YAP regulates motility, invasion, and castration-resistant growth of prostate cancer cells. Mol. Cell. Biol. 35, 1350–1362 10.1128/MCB.00102-15 25645929 PMC4372700

[316] Jiang N., Hjorth-Jensen K., Hekmat O., Iglesias-Gato D., Kruse T., Wang C. et al. (2015) In vivo quantitative phosphoproteomic profiling identifies novel regulators of castration-resistant prostate cancer growth. Oncogene 34, 2764–2776 10.1038/onc.2014.206 25065596

[317] Feng X., Degese M.S., Iglesias-Bartolome R., Vaque J.P., Molinolo A.A., Rodrigues M. et al. (2014) Hippo-independent activation of YAP by the GNAQ uveal melanoma oncogene through a trio-regulated Rho GTPase signaling circuitry. Cancer Cell 25, 831–845 10.1016/j.ccr.2014.04.016 24882515 PMC4074519

[318] Wang C., Zhu X., Feng W., Yu Y., Jeong K., Guo W. et al. (2016) Verteporfin inhibits YAP function through up-regulating 14-3-3σ sequestering YAP in the cytoplasm. Am. J. Cancer Res. 6, 27–37 27073720 PMC4759394

[319] Song S., Xie M., Scott A.W., Jin J., Ma L., Dong X. et al. (2018) A novel YAP1 inhibitor targets CSC-enriched radiation-resistant cells and exerts strong antitumor activity in esophageal adenocarcinoma. Mol. Cancer Ther. 17, 443–454 10.1158/1535-7163.MCT-17-0560 29167315 PMC5805581

[320] Morice S., Mullard M., Brion R., Dupuy M., Renault S., Tesfaye R. et al. (2020) The YAP/TEAD axis as a new therapeutic target in osteosarcoma: effect of Verteporfin and CA3 on primary tumor growth. Cancers (Basel) 12, 3847 10.3390/cancers12123847 PMC7766439 33419295

[321] Kandasamy S., Adhikary G., Rorke E.A., Friedberg J.S., Mickle M.B., Alexander H.R. et al. (2020) The YAP1 Signaling inhibitors, Verteporfin and CA3, suppress the mesothelioma cancer stem cell phenotype. Mol. Cancer Res. 18, 343–351 10.1158/1541-7786.MCR-19-0914 31732616 PMC7064165

[322] Zhang Z., Lin Z., Zhou Z., Shen H.C., Yan S.F., Mayweg A.V. et al. (2014) Structure-based design and synthesis of potent cyclic peptides inhibiting the YAP-TEAD protein-protein interaction. ACS Med. Chem. Lett. 5, 993–998 10.1021/ml500160m 25221655 PMC4160762

[323] Jiao S., Wang H., Shi Z., Dong A., Zhang W., Song X. et al. (2014) A peptide mimicking VGLL4 function acts as a YAP antagonist therapy against gastric cancer. Cancer Cell 25, 166–180 10.1016/j.ccr.2014.01.010 24525233

[324] Crawford J.J., Bronner S.M. and Zbieg J.R. (2018) Hippo pathway inhibition by blocking the YAP/TAZ-TEAD interface: a patent review. Expert Opin. Ther. Pat. 28, 867–873 10.1080/13543776.2018.1549226 30482112

[325] Pobbati A.V., Han X., Hung A.W., Weiguang S., Huda N., Chen G.-Y. et al. (2015) Targeting the central pocket in human transcription factor TEAD as a potential cancer therapeutic strategy. Structure 23, 2076–2086 10.1016/j.str.2015.09.009 26592798 PMC4660270

[326] Whitehouse M.W. (2005) Drugs to treat inflammation: a historical introduction. Curr. Med. Chem. 12, 2931–2942 10.2174/092986705774462879 16378496

